# Cost-effectiveness and potential budget impact of non-pharmacological interventions for early management in prehypertensive people: an economic evaluation for China

**DOI:** 10.1186/s12889-023-16458-1

**Published:** 2023-08-11

**Authors:** Leyi Liang, Taihang Shao, Hao Li, Mingye Zhao, Wenxi Tang

**Affiliations:** 1 Center for Pharmacoeconomics and Outcomes Research, China Pharmaceutical University, Nanjing, 211198 China; 2 Department of Public Affairs Management, School of International Pharmaceutical Business, Pharmaceutical University, 211198 Nanjing, China

**Keywords:** Prehypertension, Early management, Non-pharmacological intervention, Cost-effectiveness analysis, Budget impact analysis, China

## Abstract

**Background:**

Non-pharmacological interventions (NPIs) could be considered in the early management of prehypertensive population. This study aimed to evaluate the potential cost-effectiveness of NPIs and the budget impact of implementing NPIs on prehypertensive population in China and provide evidence of chronic disease management innovation for decision-makers.

**Methods:**

Five NPIs including usual care, lifestyle, strengthen exercise, relaxation, and diet therapy were selected based on the practice of hypertension management in China. A nine-state Markov model was constructed to evaluate the lifetime costs and health outcomes of five NPIs and a non-intervention group from the perspective of Chinese healthcare system. The effectiveness of NPIs was obtained from a published study. Parameters including transition probabilities, costs and utilities were extracted or calculated from published literature and open-access databases. Sensitivity analyses were conducted to test the uncertainty of all parameters. The impact of duration of intervention was considered in scenario analyses. A budget impact analysis (BIA) was conducted to evaluate the total cost and the medical cost saving of a hypothetical nationwide implementation of potential cost-effective NPI in prehypertensive people. Management strategies including focusing on patients with specific ages or different CVE risk levels, and different duration of implementation were taken into consideration.

**Results:**

Strengthen exercise was the most cost-effective intervention with a probability of 78.1% under the given WTP threshold. Our results were sensitive to the cost of interventions, and the utility of prehypertension and hypertension. The duration of implementation had limited impact on the results. BIA results showed that the program cost was hefty and far more than the medical cost saving with the course of simulation time. Applying management strategies which focused on individual characteristics could largely reduce the program cost despite it remained higher than medical cost saving.

**Conclusions:**

Strengthen exercise was a potential NPI that can be considered in priority for early management in prehypertensive population. Although early management can acquire medical cost saving, the related program cost can be quite hefty. Precise strategies which may help reduce the cost of early management should be taken into consideration in program design.

**Supplementary Information:**

The online version contains supplementary material available at 10.1186/s12889-023-16458-1

## Background

Prehypertension, a blood pressure (BP) category which refers to the intermediate stage between hypertension and normal BP [1], was defined as a BP range of 120–139/80–89 mmHg. However, according to the 2017 guidelines of the American College of Cardiology (ACC), BP exceeding 130/80 mmHg has been diagnosed as grade 1 hypertension [2]. Reduction of diagnostic threshold in ACC guidelines led to corresponding adjustment of hypertension management in United States. However, for countries that still use 140/90mmHg as diagnostic criteria, it indicates that BP management should be advanced to the prehypertension state. Prehypertension has a large prevalence which affects 25–50% of adults worldwide [3]. Compared with normal BP, prehypertension confers a higher risk of progression to hypertension and cardiovascular events (CVE) with a 5-year progression rate of 40% [4–6]. Therefore, adopting early management for prehypertensive population holds great value for long-term health outcomes.

Non-pharmacological interventions (NPIs) have been proven to have short-term efficacy for BP control in previous research [5, 7–11]. Meanwhile, NPIs are recommended by recent guidelines, which can be considered a priority in preventing hypertension [10, 12]. In China, more than 100 million hypertensive patients have already been covered in routine health management such as regular follow-up and physical examination [13]. Besides, NPIs such as dietary therapy, aerobic exercise, and relaxation are provided in the Primary Public Health Services for hypertension management. However, NPIs which rely on health education or behavioral changes, are highly dependent on the supervision from well-trained service providers [14–17]. As of 2021, the Chinese government had invested nearly $400 million in chronic disease management [18]. If NPIs were implemented in prehypertension in advance, there will be a huge and non-negligible expense for governmental management.

In a previously published meta-analysis, the short-term efficacy of NPIs in prehypertensive population has been proven. Combined with the meta-analysis, whether we should apply NPIs to the prehypertensive population and which NPIs are potentially cost-effective should be evaluated. Considering the scarce health resources and rising demand, it is also necessary to explore affordable service strategies under the specific NPI with cost-effectiveness. This study aimed to evaluate the potential cost-effectiveness of five representative NPIs in prehypertensive population and evaluate the input and output of implementing the potential cost-effective NPIs for early management of prehypertension in China.

## Methods

A cost-effectiveness analysis and a budget impact analysis (BIA) were conducted to accomplish the main purpose of this study. Sensitivity analyses and scenario analyses were conducted to test the robustness of the results and evaluated the impact of the duration of intervention on results in cost-effectiveness analysis. This research was reported to follow the Consolidated Health Economic Evaluation Reporting Standards (CHEERS) [19]. The protocol of this research could be found elsewhere [20].

### Interventions

According to the previously published network meta-analysis [10], we considered five NPIs: usual care, lifestyle, strengthen exercise, relaxation, and diet therapy [10]. Definitions, contents and intensities of NPIs in this study were consistent with that network meta-analysis. These NPIs were in line with the practice of BP management in China and have the potential to be applied to prehypertensive population [21]. A non-intervention group, which was also set up for comparison, was assumed to not affect BP change and have no cost. Definitions and BP reduction of these five NPIs can be found in Table 1. Detailed information on these interventions is shown in Supplementary Table 1.

**Table 1:** Definitions and BP reduction of included NPIs

Intervention	Definitions	SBP (mmHg)	DBP (mmHg)
Non-intervention	no intervention	0	0
Usual care	regular blood pressure monitoring and health education without special intervention	-0.47	-0.25
Lifestyle	comprehensive modification of lifestyle such as losing weight	-3.97	-3.11
Strengthen Exercise	different types of physical exercises under the guidance of professionals	-6.5	-3.73
Relax Exercise	physically and mental relaxation or physiotherapy such as yoga, meditation	-5.44	-5.24
Diet therapy	reasonable and regulated diet like DASH	-3.01	-1.98

Note: *SBP S*ystolic blood pressure, *DBP *Diastolic blood pressure, *DASH *Dietary approaches to stop hypertension

### Cost-effectiveness analysis

#### Study population

The initial simulated cohort was 10,000 individuals with prehypertension, of which baseline characteristics were extracted from China Health and Nutrition Surveys (CHNS). Detailed information is shown in Supplementary Table 6. CHNS is a comprehensive, long-term survey which is one of the most representative nationwide data in China [22, 23]. CHNS is created to address key public health risk factors, health outcomes, and demographic and socioeconomic aspects at individual, household, and community levels. The CHNS sought to investigate the impact of socioeconomic evolution on public health over time.

#### Model conceptualization and construction

**Fig. 1:**
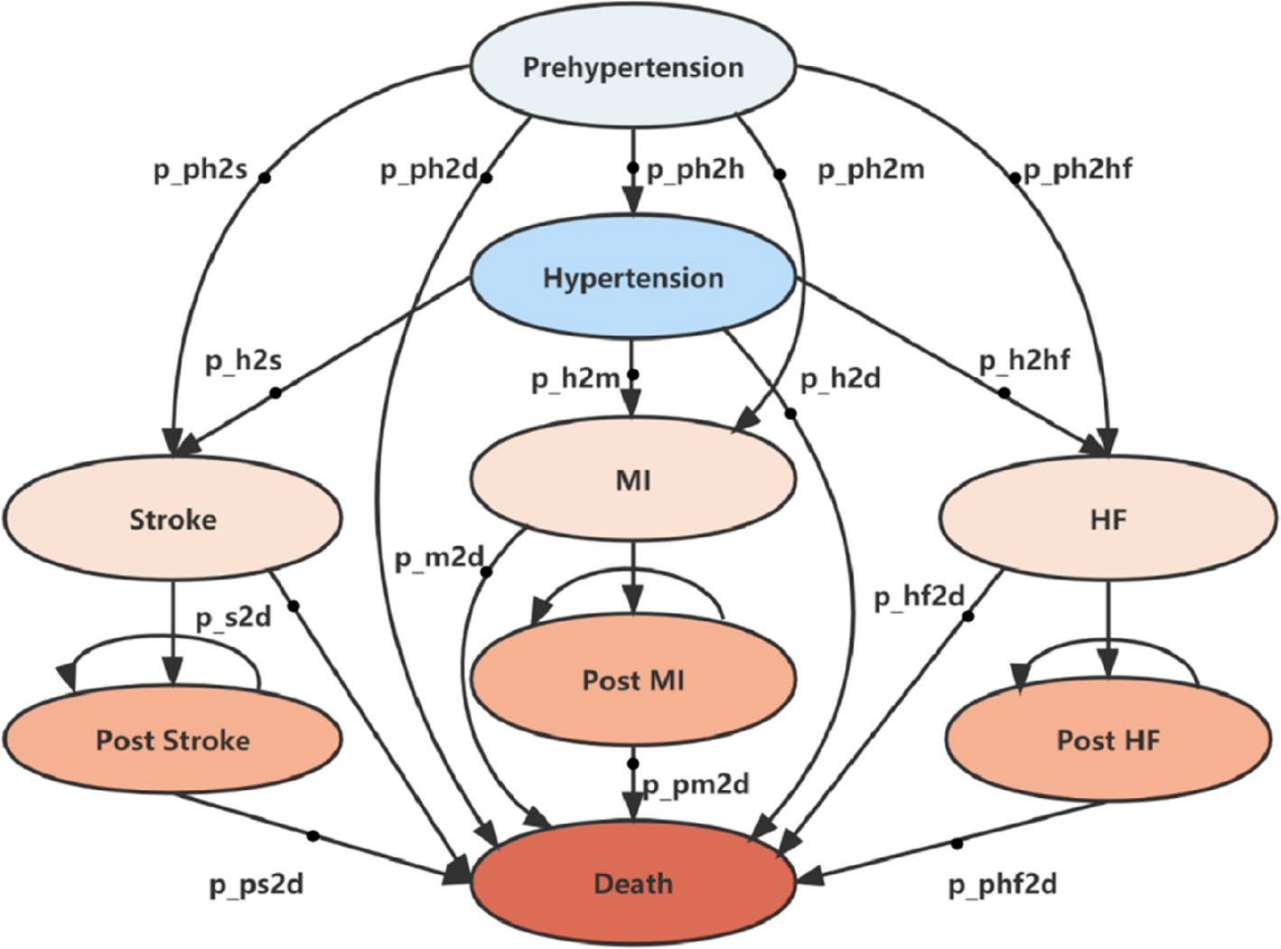
Markov model. MI: myocardial infarction; HF: heart failure

We conducted a Markov model-based cost-effectiveness analysis from the perspective of Chinese healthcare system [24, 25]. The healthcare system perspective considered the consumption of healthcare resources and the benefits to patients within the healthcare sector resulting from a particular intervention [25]. Figure 1 reflects the state-transition pathway of Markov model. The initial state was prehypertension, followed by the state of hypertension. A total of three complication states as well as their post-states (myocardial infarction, heart failure, and stroke) were considered [24]. The simulated cohort could only stay in the three complication states for one year, and would automatically progress to their post-states in the following year [24, 26]. The model used a lifetime time horizon with the cycle length set as one year to simulate the life-long disease progression of prehypertensive individuals under each NPI. The discount rate was set as 5% according to the recommendation of the “Chinese Guidelines for Pharmacoeconomics Evaluation 2020 edition“ [25]. The transition probability of prehypertension to hypertension and hypertension to each CVE state would be calculated based on risk prediction models which input the cohort characteristics such as age, gender, body mass index, etc [27–30]. Detailed information was shown in Supplementary Tables 2–6. The mortality rate of prehypertension and hypertension without CVE was considered as natural mortality rate [31]. The natural mortality was time-dependent which was derived from the Sixth National Population Census in China conducted in 2010 [31]. The transition probabilities between other health states were obtained based on existing clinical studies or mature economic models [31–36].

Model assumptions for this study could be found as follow: (1) The NPIs could effectively reduce the BP of the subjects in the first year and the effect from the second year was to maintain the BP; (2) NPIs were only applied on the prehypertension and were stopped when they leave that state; (3) All patients could only have CVE once in their lives and they could not return after entering a post-state.

We used ICER as the decision indicator, and regarded one time 2021 GDP per capita ($12,728) as the willingness to pay (WTP) threshold [37]. The financial support for NPIs, might not come from medical insurance, but the local public health funding. Therefore, a higher threshold would also be set in the sensitivity analyses (for example, three times the GDP per capita in China ($38,184)).

### Model parameters

#### Effectiveness

Since current published studies were all short-term follow-up studies, long-term outcomes including CVE could not be obtained. Therefore, we introduced BP reduction from a meta-analysis as effectiveness input [10] (Detailed BP reduction could be found in Table 1 and Supplementary Table 7). Then, age-dependent transition probabilities (from prehypertension or hypertension to CVE) were calculated through published risk prediction models [27–30]. Transition probabilities of CVE to post CVE, CVE to death, and post CVE to death were obtained from published articles.

#### Cost and utility

The cost of NPIs was calculated by decomposing them into detailed items. These items were in line with the description extracted from the published network meta-analysis [10]. For a service item that has a set price, its cost was calculated by the frequency and the unit price. We use the human capital method combined with the wages of related employees were used to calculate unpriced service items. For other items such as temporary personnel training costs, and fixed asset acquisition, the calculation method was to sum up the wages of related employees and then shared by the initial input population. The cost of each detailed item can be found in Table 2. The detailed calculation process of the cost of interventions can be found in Supplementary Tables 9, 10. Costs were updated to 2021 through Consumer Price Index [38].

For people entering the hypertension state, we considered the cost of hypertension management (Detailed items can be found in Supplementary Table 9). The cost of each CVE state was extracted from China-based studies [39, 40]. We also considered the end-of-life cost in this study, and we extracted the value from a Chinese study that targeted acute coronary syndrome [41].

Utilities of prehypertension and hypertension were extracted from a quasi-experiment in China [42]. The utilities of each CVE state were derived from other published China-based studies [43]. The detailed content of included parameters is shown in Supplementary Table 8. Quality-adjusted life-years (QALYs) were calculated by multiplying the length of time spent in a certain health state by the utility associated with that health state [26].

### Sensitivity analyses and scenario analyses

In DSA, we used the 95% confidence intervals (CI) of single effect size as the fluctuation interval. A fluctuation of 20% (considering the great uncertainty of the cost and utilities) was assumed for parameters without 95% CI. The discount rate was set to fluctuate between 0% and 8%. The results of the DSA were displayed in tornado diagrams.

We used Monte Carlo simulation with 10,000 iterations performed to do the PSA. The prior distribution of parameters was applied, such as a beta distribution for transition probability and utility, and a gamma distribution for cost. Here we also considered the uncertainty of effectiveness parameters, and a normal distribution was applied. We used scatter plots and cost-effectiveness acceptability curves (CEAC) to present the cost-effectiveness for each regimen with various WTP thresholds.

To evaluate the impact of the duration of NPIs intervention on base-case analysis results, we further considered several scenario analyses, in which the years of NPIs implementation varied from one year to the lifetime time horizon.

### Budget impact analysis

A BIA model was constructed to estimate the program cost and the medical cost saving of national implementation of potential cost-effective NPI for prehypertensive population from the perspective of Chinese healthcare system. The program cost was calculated by multiplying the implementation cost with the Chinese demographic characteristics [44]. Medical cost saving was considered as the reduction expenditure in the medical costs of health states followed by prehypertension compared to non-intervention. The simulation time was set as 15 years. We also included scenarios of different early management strategies, which considered three factors: (1) the beginning age of early management (Four age groups were considered: “45–49”, “50–54”, “55–59”, “60–64”); (2) whether to include only patients with high risk of CVE into early management; (3) duration of implementation varied from one year to simulation horizon. In addition, compliance to NPIs including 100%, 80%, 60%, 40%, 20% was also considered in this model. Details of key model assumptions, methodologies, and parameters input are shown in the Supplementary material p18-21.

**Table 2:** Cost table of detail items

Item	Cost for each patient (price and frequency)
***Early hypertension management*** [21]	
Examination revealed	1.57 USD/time, 4 times a year
Follow up	4.08 USD/time, 4 times a year
Health examination	2.67 USD/time, 4 times a year
Health education (Lecture based)	1.88 USD/time, 2 times a year
Total Cost	37.02 USD
***Usual Care*** [21]	
Health education (Lecture based)	1.57 USD/time, 2 times a year
Regular blood pressure monitoring (Follow up)	4.08 USD/time, 4 times a year
Total Cost	20.08 USD
***Lifestyle***	
Management staff [45]^a^	88.39 USD/year
Daily education	0.05 USD/day, everyday
Early hypertension management	37.02 USD/year
Total Cost	142.59 USD
***Strengthen Exercise***	
Training cost of staff*^b^	3923.13 USD/year
Management staff	88.39 USD/year
Isometric exercise	3.14 USD/time, 3 times/week, 8 weeks a year
Aerobic exercise	4.08 USD/time, 3 times/week, 8 weeks a year
Including whole body muscle strength training, motion training of each joint, freehand gymnastics, equipment training, gait balance function training	2.04 USD/time, 3 times/week, 8 weeks a year
Treadmill [46, 47]#	0.95 USD/year
Resistance band	0.09 USD/year
Early Hypertension management	37.02 USD/year
Total Cost	200.50 USD
***Diet therapy***	
Nutrition plan and training for dietitians [48]*^c^	14118.75 USD/year
Average annual consumption expenditure for ordinary diet [49, 50]^d^	883.38 USD/year
Average annual consumption expenditure for DASH [49, 50]	3.77 USD/day, everyday
Early Hypertension management	37.02 USD/year
Total Cost	527.87 USD
***Relaxation***	
*Yoga* [51]	
Yoga Training cost*#^e^	8409.10 USD/year
Yoga Training course production cost*#^e^	8409.10 USD/year
Yoga Service charge#	15.02 USD/class, 8 classes
Yoga mat	0.14 USD/year
*Acupuncture* [52–54]	
Physiotherapist salary#	178.06 USD/year
Acupuncture	3.61 USD/time, 3 times/week, 8 weeks a year
*Meditation* [55]	
Physiotherapist appointment	7.80 USD/appointment, 8 appointments in total
Meditation	48.74 USD/session, 8 sessions in total
Management staff	88.39 USD/year
Early Hypertension management	37.02 USD/year
Total Cost	404.49 USD

*The cost is only calculated once in the first year and is the program design cost for each intervention; # The cost is discounted; ^a^ Annual salary of Management staff; ^b^ Annual salary of rehabilitation department; ^c^ Annual salary of a dietitian; ^d^ The annual per capita food consumption expenditure of Chinese residents reaches; ^e^ Annual salary of a yoga teacher

## Results

### Cost-effectiveness analysis

Results of base-case analysis are shown in Table 3. It showed that relaxation and diet therapy had greater costs but fewer QALYs gained, which were strongly dominated by other interventions. Usual care and lifestyle were extended dominated by strengthen exercise. Details were shown in the cost-effectiveness plane(Supplementary Fig. 3). After ruling out all dominated interventions, strengthen exercise was cost-effective compared to non-intervention, given that the ICER was lower than the WTP threshold of $12,728 per QALY.

According to the breakdown results of the base-case analysis (shown in Supplementary Tables 11, 12), the differences in costs and QALYs gained in prehypertension and hypertension state might contribute more to the differences between the results. We also found that strengthen exercise had the longest average time for people from prehypertension transition to hypertension, followed by relaxation and lifestyle, which was in line with the effectiveness indicators.

**Table 3:** Results of base-case analysis

	Trans	Costs	QALYs	ΔCosts	ΔQALYs	ICER
A (all intervention)						
Non-intervention	7.22	3082.28	12.95	/	/	/
Usual care	7.36	3180.21	12.97	97.93	0.02	6037.46(E.D)
Lifestyle	8.62	3842.79	13.11	662.58	0.14	4741.07(E.D)
Strengthen exercise	9.39	4223.36	13.19	380.57	0.09	4429.33
Relaxation	9.29	5683.48	13.18	1460.12	-0.01	-131236.98
Diet therapy	8.22	6352.31	13.06	668.83	-0.12	-5627.03
B (excluding dominated intervention)						
	Trans	Costs	QALYs	ΔCosts	ΔQALYs	ICER
Non-intervention	7.22	3082.28	12.95	/	/	/
Strengthen exercise	9.39	4223.36	13.19	1141.08	0.24	4622.38

Note: *Trans*: the average time for people from prehypertension transitioned to hypertension; *ICER*: incremental cost-effectiveness ratio; Unit: *USD*; *E.D*: extended dominated

All interventions are ranked in order of cost from smallest to largest. The presented ICERs are calculated by two adjacent interventions

**Fig. 2:**
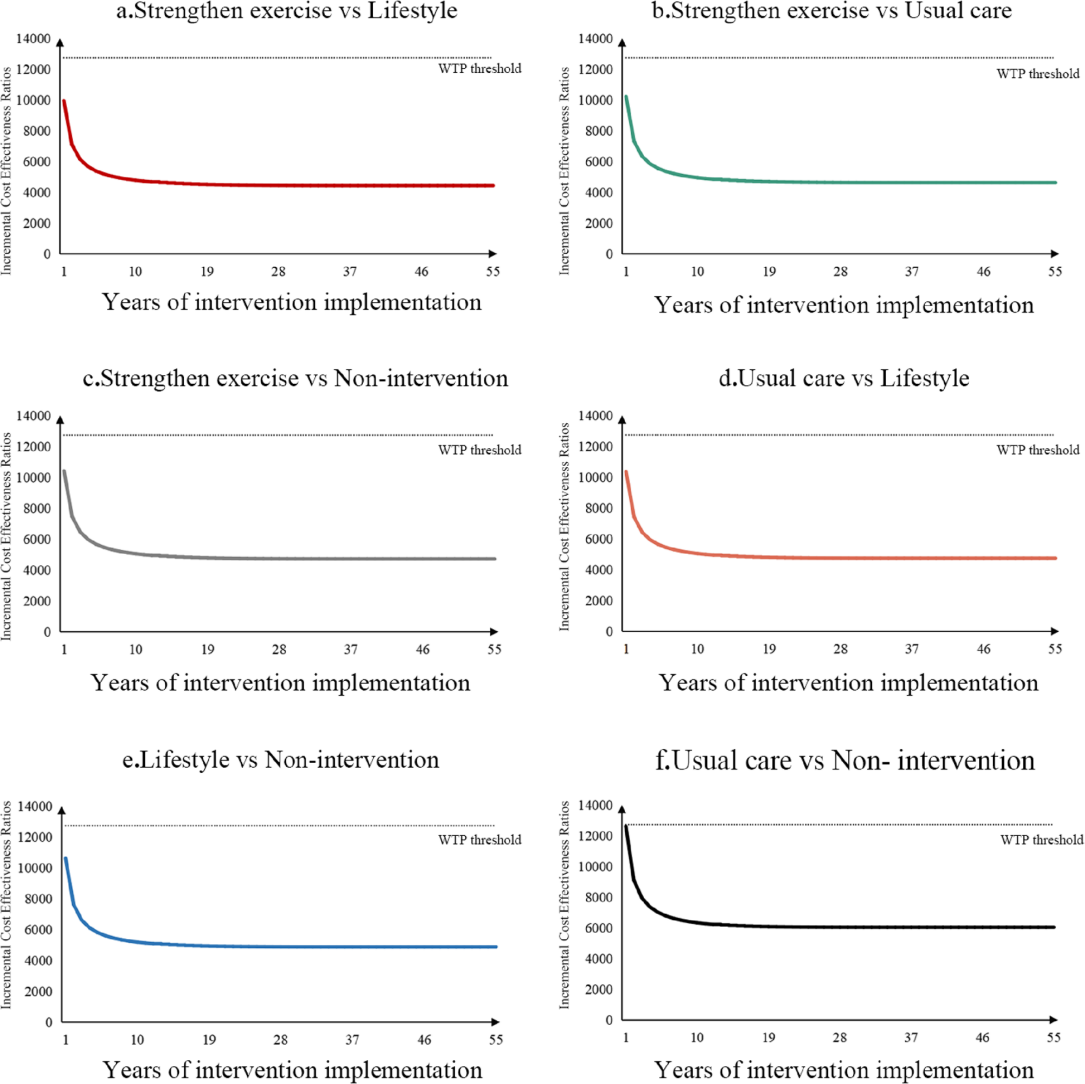
Results of scenario analyses

The results of scenario analyses are shown in Fig. 2. Relaxation and diet therapy were excluded from the scenario analyses since they were strongly dominated by other interventions in the base-case analysis. With the years of implementation increasing to seven, the ICERs between all groups dropped consistently from their highest point. After years of implementation increasing to ten, the ICERs between all groups became steady. The ICERs between usual care and non-intervention were higher than in other comparisons. Although ICER between usual care and non-intervention with one year of implementation reached $12,615, which was the highest among all scenarios, it was still lower than the WTP threshold($12,728).

### Sensitivity analyses

Results of DSA and PSA are shown in Supplementary Fig. 4a and b. We included totally four comparisons in DSA. The results were sensitive to utilities of prehypertension and hypertension in all comparisons. Cost of interventions for prehypertension and discount rate also influenced the results. Note that the fluctuation in the utility of hypertension could even lead to the change in the conclusions of all four comparisons. This was further discussed in the discussion.

**Fig. 3:**
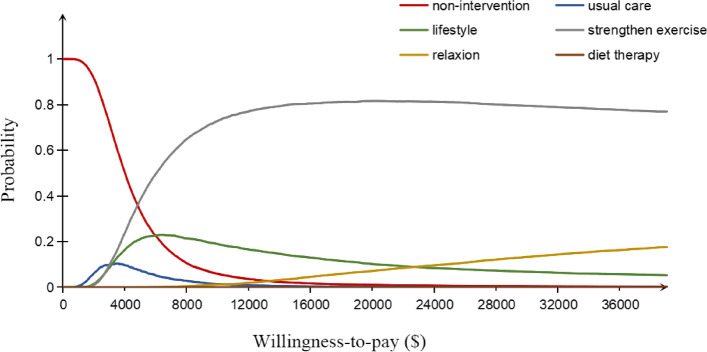
Probabilistic Sensitivity analyses: cost-effectiveness acceptability curve (10,000 iterations). WTP: willingness-to-pay; CEAC: cost-effectiveness acceptability curve

The cost-effectiveness acceptability curve is shown in Fig. 3. Non-intervention had the highest probability to be cost-effective when WTP ranged from 0$ to $4,700. At a WTP threshold of $12,728 per QALY, strengthen exercise had a probability of over 78.1% being cost-effective. This probability rose to 77.1% when WTP threshold reached $38,184 per QALY.

### Budget impact analysis

**Fig. 4:**
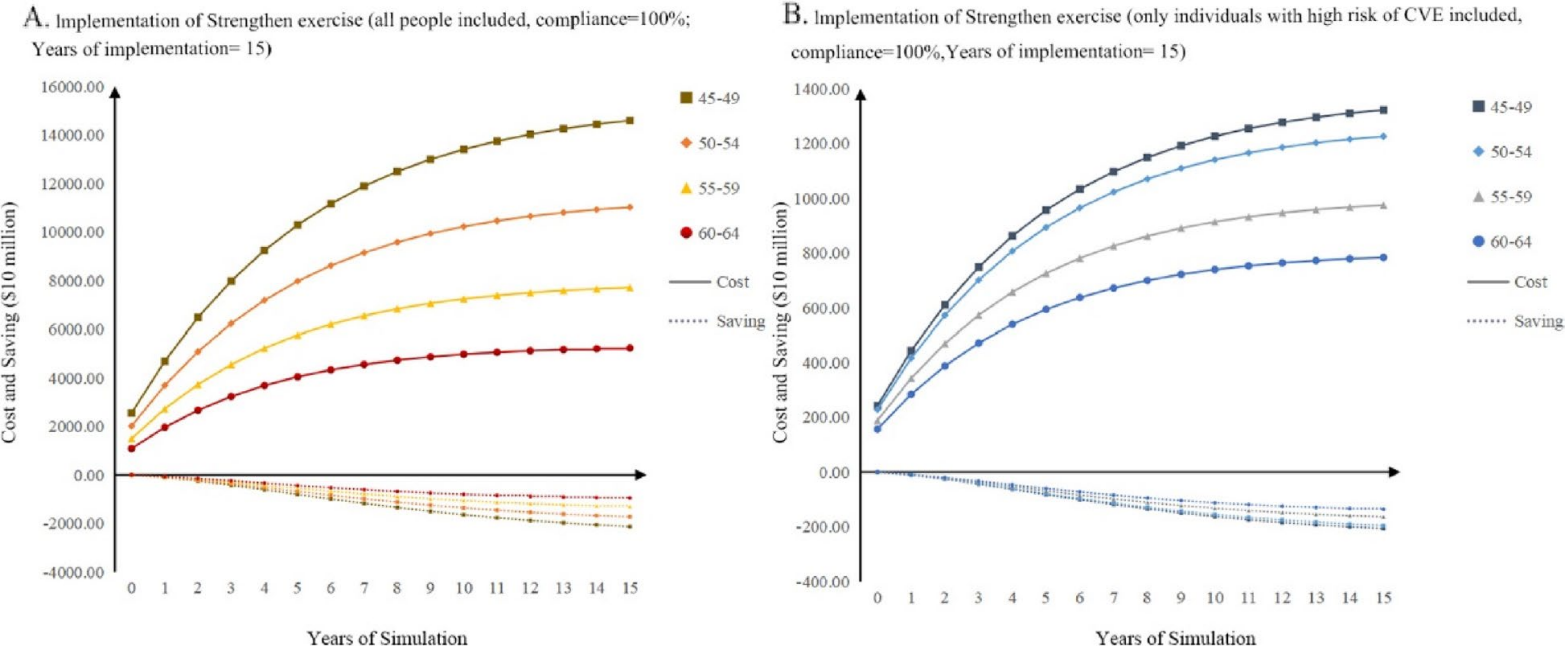
Results of Budget impact analysis results

According to the base-case results, we only considered strengthen exercise in the budget impact analysis. Results of budget impact analysis of which the duration of implementation is 15 years are shown in Fig. 4. The medical cost saving, which increased with the course of simulation time,was far less than the hefty program cost. Through considering some specific strategies including decreasing the duration of intervention, starting intervention in older population, and only managing population with high risk of CVE, we could found that the previous conclusion was unchanged. However, we found that through these strategies, the program costs could be reduced. In addition, according to Fig. 4, changing a strategy might lead to little change in cost saving, but huge reduction in program costs could be observed. (Detailed results can be found in the Supplementary Fig. 5a, b, c and d).

## Discussion

According to the results of base-case analysis, strengthen exercise might be the most cost-effective intervention at the WTP threshold of one-time GDP per capita in China. DSA results showed that our results were sensitive to the utility of prehypertension and hypertension, discount rate, and the costs of interventions. PSA results showed that when the WTP was set to one-time GDP per capita, strengthen exercise had the highest probability to be cost-effective. The other interventions were not associated with a probability of greater than 50% being cost-effective. Implementation of NPIs within seven years had great impact on the results. However, results become stable when years of implementation over ten. BIA showed that the program implementing strengthen exercise for early intervention in prehypertensive individuals could bring medical cost saving. However, the program cost was hefty and far more than the medical cost saving. Focusing on patients with specific characteristics or shortening years of implementation can help reduce the program cost.

Drug interventions were not considered in this cost-effectiveness analysis, since there were already two high-quality studies discussed the use of antihypertensives in the Chinese prehypertensive people. Zhou et al. developed a microsimulation model to compare costs and effectiveness of drug treatment and NPIs for prehypertensive people over a lifetime horizon from a government affordability perspective [17]. Their results showed that at a WTP threshold of one time GDP per capita in 2017, drug interventions only had a 1.8% probability of being cost-effective compared with NPIs. Therefore, they suggested that drug treatment was not cost-effective compared with NPIs for target population. Chen et al. built a Markov state-transition model to simulate a hypothetical cohort of Chinese adults with high-range prehypertension but without CVE [24]. They found that compared with placebo, drug treatment had great benefits in delaying the development of hypertension but was far from cost-effective at a WTP threshold of one time GDP per capita in 2014. Therefore, considering the current existed evidence, we did not included the drug interventions in our study. However, there were lack of current studies which targeted on the cost-effectiveness of NPIs for prehypertensive people. Thus, our study results could make up for this deficiency.

According to DSA results, for all four comparisons, the utility of prehypertension and hypertension, and cost of interventions had great impacts on our results. This could also be identified from the breakdown of base-case analysis. Costs and QALYs gained in prehypertension and hypertension states differed greatly between groups. It is also notable that the fluctuation of the utility of hypertension could even lead to the change in conclusion when WTP was set as $12,728. However, when WTP was set as $38,184, our conclusion was stable. Utility of prehypertension and hypertension used in this study, which was based on Guo’s study [42], is 0.931 and 0.8 respectively. Guo conducted a quasi-experiment in China to investigate the effectiveness of a non-pharmacological intervention on the prehypertensive population, and the utility of different states of BP conversion process was measured. Therefore, we considered that these two values could be used in our China based study. Some similar study which focused on prehypertensive people made an assumption that utility of prehypertension and utility of hypertension were the same [17, 24]. However, we did not believe that this assumption was reliable. Spruill’s study found that people with prehypertension had nearly the same quality of life as normal people [56], while Wang’ s study found that people with hypertension had significantly different quality of life from normal people [57]. These two indirect evidence showed that the utility of prehypertension and hypertension states can be different. Therefore, in our study, we considered different utility values for these two states. Nevertheless, due to the high uncertainty of these two utility values in our study, future studies targeted on the the utilities of prehypertensive and hypertensive people that are representative of China are needed to resolve this parameter uncertainty. For costs of interventions, which were calculated from included studies, might be subjective. However, this uncertainty was hard to handle since it was difficult to find an authoritative reference. Thus, a national guideline to instruct management staff to implement NPIs was needed to improve the calculation of related costs.

Some NPIs were proven to be cost-effective in CEA, however, the results of BIA show the affordability of the implementation program was concerning. According to hypertension guidelines from each country, there seems a radical approach to including people with prehypertension in management. Although 2017 ACC guidelines have made this attempt, the implementation of early management in low- and middle-income countries still needs further consideration. Because according to our BIA results, the program cost could be a huge expense and greatly exceeded the medical cost saving in this BIA. However, the medical cost saving was underestimated because not only individuals with prehypertension can benefit from the program. Our research only focuses on the CVE related to hypertension, therefore, other chronic diseases related to comorbidity which may produce extra medical costs were not involved. For example, some previous research proved that NPIs have potential effectiveness in diseases such as prediabetes [58, 59]. This suggested people with prehypertension or prediabetes might benefit from health service innovation like integrated care. In addition, in this BIA, we explored several potential management strategies to give more precise interventions. These strategies including setting the beginning age of early management, including only patients with high risk of CVE, and reducing the duration of implementation, can all help to reduce the program cost through managing people more accurately. Although the program cost remained high, it could be largely reduced if the interventions based on individual characteristics were applied, which could only lead to little change in cost saving. This can be an inspiration to future research which focused on precise chronic disease prevention. The changes of input and output of different strategies may provide decision-makers with more information to choose the optimal management strategy.

However, there are several limitations in the study. Firstly, NPIs included in this study were all extracted from current published studies and guidelines. Since the costs were calculated subjectively, some currently unsolvable biases might be introduced to our results. Secondly, effectiveness parameters were obtained from a network meta-analysis which based on global population, their generalization of to the Chinese context needed further assessment. Thirdly, utility values of CVE related states used in this study were extracted from different sources. There might exist biases that the utility gap between health states was not fully attributed to changes in utility due to changes in health status. Thus, we considered a 20% upper and lower range of fluctuations in DSA, and the results showed that the conclusions were stable. Fourth, the transition probabilities between CVE related states in our model could be quite similar among different NPIs and had no direct relationship with NPIs. Besides, our model assumed that only in the first year could the NPIs effectively reduce the BP of the subjects and the effect from the second year was to maintain the BP. These two could be strong assumptions without evidence support. However, since follow-up of current NPIs studies mainly did not exceed one year, long-term BP benefits could not be obtained from current evidence. Therefore, this limitation may be handled by further high-quality long follow-up studies. Fifth, we used the static cohort in BIA rather than the dynamic cohort, which might lead to imprecise estimation. Therefore, we only dropped the qualitative conclusions instead of providing quantitative outcomes.

## Conclusion

Implementing NPIs in prehypertensive population is a recommended management scheme. According to our research, strengthen exercise was potentially cost effective which can be considered in priority for early management in prehypertensive population. Although early management can acquire medical cost saving, the related program cost can be quite hefty. Precise strategies which may help reduce the cost of early management should be taken into consideration in program design.

### Supplementary Information

**Additional file 1.**

## Data Availability

All data generated or analyzed during this study are included in this published article and supplementary files. The unpublished network meta-analysis is available from the corresponding author with reasonable request.

## Code availability

R codes for this study are available on GitHub

( https://github.com/TaihangShao/prehypertension_CEA_model ).
