# Supplementary material for: Cost-effectiveness and potential budget impact of non-pharmacological interventions for early management in prehypertensive people: an economic evaluation for China
Source: BMC Public Health. 2023 Aug 11;23:1531. doi: 10.1186/s12889-023-16458-1 (PMC10416408; doi:10.1186/s12889-023-16458-1)
Supplement: Supplementary file 1 — Additional file 1. [file 12889_2023_16458_MOESM1_ESM.docx]

**Supplementary material**

## Cost-Effectiveness and Potential Budget Impact of NPIs for Early Management in Prehypertensive People: An economic Evaluation for China

Leyi Liang^1*^, Taihang Shao^1*^, Hao Li^1^, Mingye Zhao^1^, Wenxi Tang^1,2#^

^1^ Center for Pharmacoeconomics and Outcomes Research, China Pharmaceutical University, Nanjing, China, 211198

^2^ Department of Public Affairs Management, School of International Pharmaceutical Business, China Pharmaceutical University, Nanjing, China, 211198

*These authors contributed equally to this work.

#Correspondence:

Wenxi Tang

E-mail: tokammy@cpu.edu.cn

**Content**

[1. Non-pharmacological interventions (NPIs) for prehypertensive population 3](#_Toc137664741)

[2. Effect parameter conversion 4](#_Toc137664742)

[2.1 Prediction of hypertension risk 4](#_Toc137664743)

[2.2 Prediction of stroke risk 4](#_Toc137664744)

[2.3 Prediction of heart failure risk 4](#_Toc137664745)

[2.4 Prediction of myocardial infarction risk 5](#_Toc137664746)

[2.5 Include risk prediction analysis group 5](#_Toc137664747)

[2.6 Specific parameter conversion process 6](#_Toc137664748)

[2.7 Results of single-arm meta-analysis 6](#_Toc137664749)

[2.8 A Limitation which can not be ignored and related solutions 8](#_Toc137664750)

[3. Parameters input of the Markov model 9](#_Toc137664751)

[4. Calculation of the Cost of Interventions 11](#_Toc137664752)

[4.1 Cost calculation of usual care 11](#_Toc137664753)

[4.2 Cost calculation of lifestyle 11](#_Toc137664754)

[4.3 Cost calculation of strengthen exercise 11](#_Toc137664755)

[4.4 Cost calculation of relaxation 12](#_Toc137664756)

[4.5 Cost of diet therapy 12](#_Toc137664757)

[5. Results for base-case analysis 15](#_Toc137664758)

[5.1 Breakdown results of base-case analysis 15](#_Toc137664759)

[5.2 The cost-effectiveness plane of base-case analysis 16](#_Toc137664760)

[6. Sensitivity Analyses Plot 17](#_Toc137664761)

[6.1 Deterministic sensitivity analysis 17](#_Toc137664762)

[6.2 Probabilistic sensitivity analysis 17](#_Toc137664763)

[7. Key model assumptions, methodologies and parameters input of BIA 17](#_Toc137664764)

[7.1 Key model assumptions of BIA 17](#_Toc137664765)

[7.2 Methodologies 18](#_Toc137664766)

[7.3 Parameters input 18](#_Toc137664767)

[7.4 Scenario analyses of Budget Impact Analysis 19](#_Toc137664768)

[References 21](#_Toc137664769)

## 1. Non-pharmacological interventions (NPIs) for prehypertensive population

These standard NPIs were extracted from a published network meta-analysis (NMA). In this NMA, the content of these intervention were also extracted and summarized from included studies and guidelines. This NMA was available from the corresponding author with reasonable request.

| Supplementary Table1 NPIs for prehypertensive population | | |
| --- | --- | --- |
| **Name** | **Content** | **Intensity** |
| **Non-intervention** | no intervention | / |
| **Relaxation** | Receive physical therapy by professionals. Or receive training by professionals, learn relaxing exercises, combine learning materials for independent exercises, including yoga, meditation. | Average 4 times/week, 45 minutes each time, lasting 12 weeks |
|  |  |  |
|  |  |  |
| **Strengthen Exercise** | Under the guidance of professionals, perform aerobic exercise (such as jogging, brisk walking, cycling, etc), resistance exercise, isometric exercise or a combination of multiple forms of exercise at different target intensities. | Average 4 times/week, 45 minutes each time, lasting 8 weeks |
|  |  |  |
|  |  |  |
|  |  |  |
|  |  |  |
| **Dietary** | Reasonable diet, reduce sodium intake, reduce fat intake, reduce alcohol, increase potassium intake or adopt DASH diet. | Adhere to certain diet every day. Conduct education every certain times. |
|  |  |  |
|  |  |  |
|  |  |  |
| **Lifestyle** | Participants are required to change lifestyle or lose weight according to education. | Everyday |
|  |  |  |
| **Usual care** | Regular blood pressure monitoring and health education is conducted without specific intervention. | Follow up once a month and conduct health education once every six months |

## 2. Effect parameter conversion

Since almost none of the included studies reported the incidence of cardiovascular events (CVE), this study used the reduction of blood pressure (BP) as the effect indicator. Since the reduction of BP cannot be directly substituted into transition probability in Markov model, we brought a risk prediction model to calculate the transition probability of four states (hypertension, stroke, myocardial infarction, and heart failure)^1-4^. We used the cross-sectional data from the Chinese Health and Nutrition Survey (CHNS) to extract the parameters required in the risk prediction model. Then we input them into the risk prediction model for calculation to make the predicted transition probability closer to the Chinese population characteristics.

### 2.1 Prediction of hypertension risk

The hypertension risk prediction model came from Chien’s study^1^. This 12-year cohort study begun in Taiwan followed a total of 2506 patients. Weibull regression was adopted to construct a biochemical model and a clinical model. We used the biochemical model in our study. The required parameters were Sex, Age, BMI, SBP, DBP, WBC, FG and UA. The specific regression coefficient table is shown in the following table:

| Supplementary Table 2 Regression coefficients from Chien’s study | | | | | | | | | | |
| --- | --- | --- | --- | --- | --- | --- | --- | --- | --- | --- |
| Name | Sex | Age | BMI | SBP | DBP | WBC | FG | UA | intercepts | weibull scale |
| Label | β1 | β2 | β3 | β4 | β5 | β6 | β7 | β8 | α | lamda |
| Value | 0.037 | -0.01 | -0.036 | -0.028 | -0.013 | -0.035 | -0.001 | -0.038 | 8.604 | 0.589 |

The calculation formula of risk probability *P* is as follows:

$$P=1-exp(-exp(\frac{Ln(t)-\sum_{i=1}^{n} \beta_{i}X_{i}}{\sigma}))$$

（ *n* is the number of parameters, $\sigma$ is the Weibull Scale parameter, *t* is the time to predict risks.）

### 2.2 Prediction of stroke risk

Stroke risk prediction models were derived from Chien's study^3^. This study used the same cohort data as the hypertension risk prediction Model^1^. This study used a COX proportional risk model, and a biochemical model and a clinical model were built. We adopted the biochemical model and parameters needed included Age, Sex, SBP, DBP, family history, AF, TC, WBC, and FG. The specific regression coefficients are shown in the following table:

| Supplementary Table 3 Regression coefficients from Chien’s study | | | | | | | | | |
| --- | --- | --- | --- | --- | --- | --- | --- | --- | --- |
| Name | Age | Sex | SBP | DBP | family history | AF | TC | WBC | FG |
| Label | β1 | β2 | β3 | β4 | β5 | β6 | β7 | β8 | β9 |
| Value | 0.073 | -0.458 | 0.016 | 0.017 | 0.457 | 1.247 | 0.003 | 0.081 | 0.005 |
| Mean | 54.6 | 1.53 | 125.1 | 77 | 0.2 | 0.01 | 197.8 | 6.3 | 109.9 |

A 10-year basic risk function *S(10)*=0.9783.

The 10-year stroke risk prediction function is as follows:

$$h(t)=h_{0}(t)exp\{\beta^{'}X-\beta^{'}X^{-}\}$$

*X*^-^ is the mean value parameter of the model.

### 2.3 Prediction of heart failure risk

The heart failure risk prediction model came from Kannel's study which followed 486 patients for up to 38 years^2^. Logistic regression was used to predict the incidence of male and female reports by gender. The male model was used in our study (Some parameters cannot be obtained in the female model). Required parameters were Age, LVH, heart rate, SBP, CHD, VD, DM and BMI. The specific regression coefficient table is shown in the following table:

| Supplementary Table 4 Regression coefficients from Kannel's study | | | | | | | | | |
| --- | --- | --- | --- | --- | --- | --- | --- | --- | --- |
| Name | Age | LVH | heart rate | SBP | CHD | VD | DM | BMI | intercepts |
| Label | β1 | β2 | β3 | β4 | β5 | β6 | β7 | β8 | α |
| Value | 0.0503 | 1.3402 | 0.0105 | 0.00337 | 1.5549 | 1.3929 | 1.3857 | 0.0578 | -10.7988 |

The model predicted 4-year risk of heart failure events(*f(x)*)：

$$f(x)=\frac{1}{1+e^{-g(x)}}$$

$$g(x)=\omega_{0}+\omega_{1}X_{1}+\ldots+\omega_{k}X_{k}$$

### 2.4 Prediction of myocardial infarction risk

The myocardial infarction risk prediction model was derived from Zhang's research that followed a cohort of 5092 patients from 1974 to 1993^4^. This study adopted COX risk proportion model. The required parameters were Age, SBP, TC, BMI, and smoker proportion. Specific regression coefficients are shown in the following table:

| Supplementary Table 5 Regression coefficients from Zhang's study | | | | | | |
| --- | --- | --- | --- | --- | --- | --- |
| Name | Age | SBP | TC | BMI | smoker | intercepts |
| Label | β1 | β2 | β3 | β4 | β5 | α |
| Value | 0.040129 | 0.0229901 | 0.0105563 | 0.1269031 | 0.8426276 | -7 |

A 10-year basic risk function *S(10)*=0.9997.

The 10-year stroke risk prediction function is as follows:

$$h(t)=h_{0}(t)exp\{\beta^{'}X\}$$

*X* is the mean value parameter of the model. In this study, the exponential part of the model was adjusted, so it didn’t need the mean value and can be directly calculated by multiplying the substitution value by the regression coefficient.

### 2.5 Include risk prediction analysis group

The data included in this study were cross-sectional data from the CHNS survey (https://www.cpc.unc.edu/projects/china/data/datasets/index.html). The 2009 cross-sectional coincident population (n=7785) was extracted from the long-term follow-up data sets PE-PA and Bio. After clearing the missing data, 5934 people remained. After further processing, we included 2126 prehypertensive people and 1066 hypertensive people. The baseline data were averaged and the unreported data needed by the model were extracted from studies with the Chinese population as the research object. The baseline data used in the risk prediction model was shown in the :

| Supplementary Table 6 Baseline data used in the risk prediction model | | |
| --- | --- | --- |
|  | Prehypertension(n=2126) | Hypertension(n=1066) |
| **Men%** | 45% | 45% |
| **age** | 46.88 | 46.88 |
| **BMI** | 24.01 | 24.99 |
| **sbp** | 126.63 | 156.21 |
| **dbp** | 81.8 | 92.48 |
| **WBC** | 6.25 | 6.32 |
| **FG** | 99.91 | 106.94 |
| **UA** | 5.07 | 5.55 |
| **family history**^5^ | 34.90% | 40.4% |
| **AF**^6^ | 1.03% | 1.03% |
| **TC** | 192.14 | 201.22 |
| **DM%** | 2.68% | 6.60% |
| **smoking%** | 31.33% | 31.52% |
| **CHD%** | 0.42% | 2.63% |
| **LVH**^7^ | 1.80% | 4.6% |
| **heart rate**^8^ | 78 | 78 |
| **VD**^9^ | 0.40% | 0.40% |

### 2.6 Specific parameter conversion process

The relative effect of each intervention compared with lifestyle can be obtained through the league table after the NMA. Then, we use a single-arm meta-analysis to summarize the absolute effect of Lifestyle. We obtain the absolute effect of other interventions through. Combining these absolute effects with the BP of the risk prediction population can approximately obtain the BP after receiving each intervention. It is assumed that NPIs have no effect on other outcome indicators of this.

Under the situation of each intervention, substituting the BP and other baseline data into the risk prediction model can obtain the risk of hypertension, stroke, myocardial infarction and heart failure in the Chinese prehypertensive population. Bringing the data of the population with hypertension into the risk prediction model can approximately calculate the risk of hypertension, stroke, myocardial infarction and heart failure in the Chinese hypertensive population. After obtaining the probability of occurrence of an event in a period of time (such as a 10-year risk), it needs to be transformed into a transition probability in a period (1 year):

$$r=-\frac{ln(1-p_{1})}{t_{1}}$$

$$p_{2}=1-exp(-rt_{2})$$

Where, *r* is the instantaneous incidence rate, *p_1_* is the 10-year risk, *p_2_* is the probability of occurrence of an event within a cycle, *t_1_* is 10 years, *t_2_* is 1 year.

Since this study intends to obtain the transition probability of the population after the age of 45, data of population level are substituted in the model instead of individual data (each individual has a different age). Therefore, in sensitivity analysis, we will adopt a larger fluctuation to explore the impact of transition probability on the results. Since the related transition probability was considered to change by age, the probability of these risk events will be presented in the model in the form of lifetable.

### 2.7 Results of single-arm meta-analysis

Both SBP and DBP included twenty-two studies into the single-arm meta-analysis in Lifestyle (excluding those studies comparing Lifestyle and Usual Care). Finally, the SBP reduction of Lifestyle was 2.26mmHg, the DBP reduction of Lifestyle was 1.65mmHg. As shown in the figures below.

| 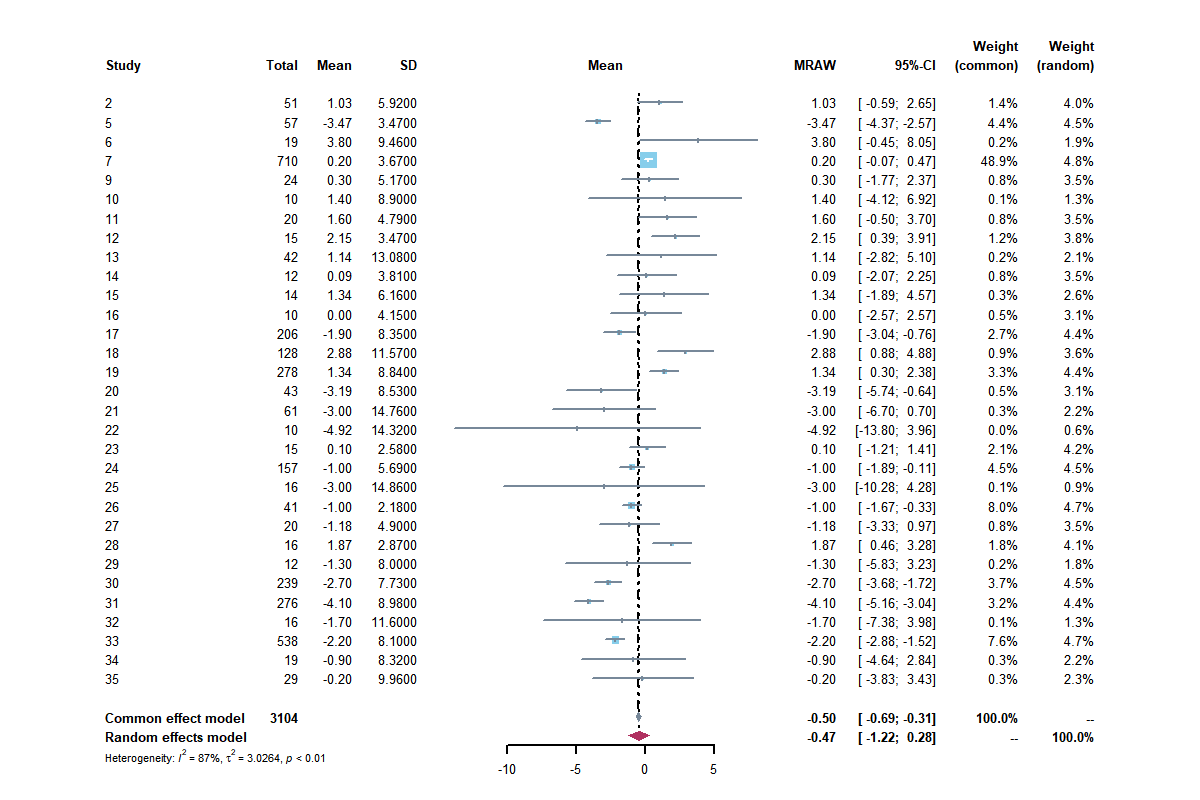 |
| --- |
| Supplementary Figure 1 SBP reduction of Usual Care |
| 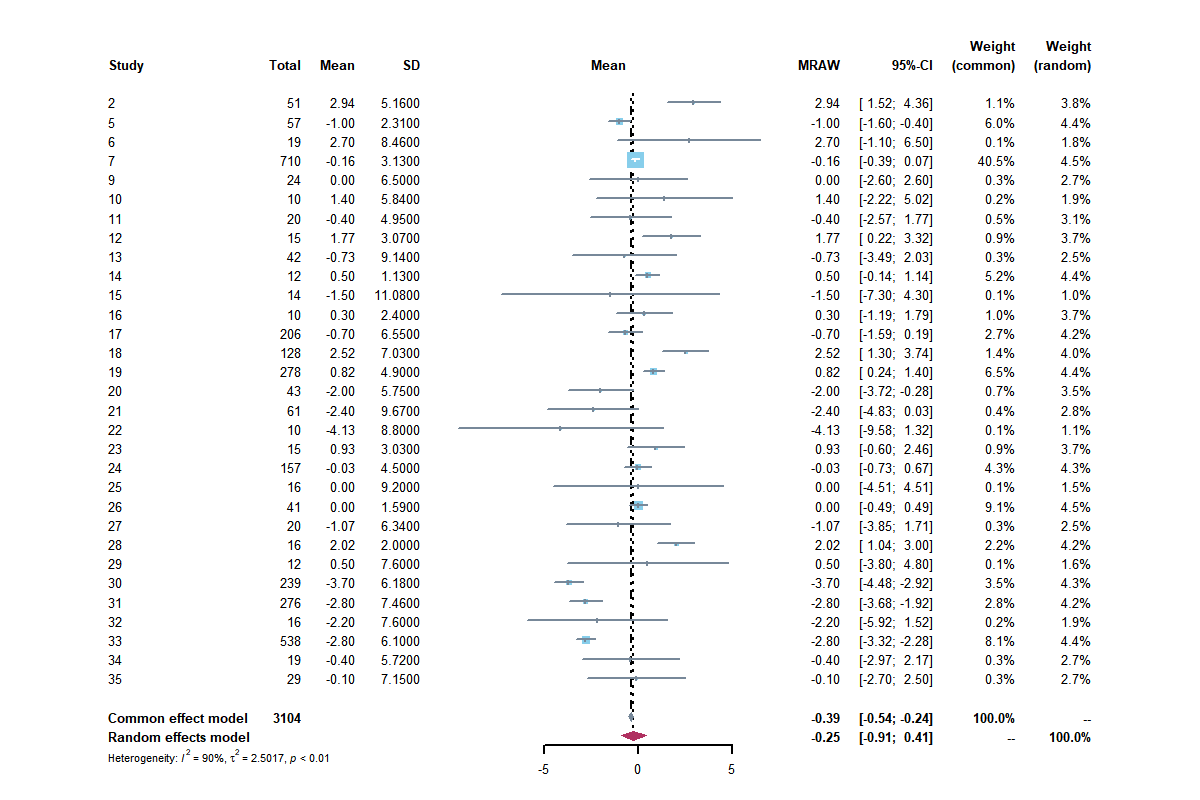 |
| Supplementary Figure 2 DBP reduction of Usual Care |

The absolute BP reduction of each interventions can be obtained by combining the results of single-arm meta-analysis with the relative BP reduction in league table. Absolute BP reduction are shown in the table below.

| Supplementary Table 7 Absolute BP reduction of five NPIs | | |
| --- | --- | --- |
| Intervention | SBP (mmHg) | DBP (mmHg) |
| Usual care | -0.47 | -0.25 |
| Lifestyle | -3.97 | -3.11 |
| Strengthen Exercise | -6.5 | -3.73 |
| Relax Exercise | -5.44 | -5.24 |
| Diet therapy | -3.01 | -1.98 |

### 2.8 A Limitation which can not be ignored and related solutions

A limitation which could not be ignored was the uncertainty of effectiveness input. The NMA we used was calculated from a global perspective. This meant that the BP reduction effects might differ from the Chinese local effects. Besides, the risk prediction models used in this study were all individual based, which might lead to misleading estimation when we used a cohort as the input. Therefore, we considered the 95% CI of BP reduction value in the NMA when conducting the sensitivity analysis to test the uncertainty of effectiveness input.

## 3. Parameters input of the Markov model

| Supplementary Table 8 Parameters input of the Markov model | | | | | | |
| --- | --- | --- | --- | --- | --- | --- |
| **Name** | **Description** | **Value** | **Lower** | **Upper** | **Distribution** | **Source** |
| **Tran** |  |  |  |  |  |  |
| p_ph2h | prehypertension to hypertension | lifetable |  |  |  |  |
| p_ph2s | prehypertension to stroke | lifetable |  |  |  |  |
| p_ph2m | prehypertension to MI | lifetable |  |  |  |  |
| p_ph2hf | prehypertension to heart failure | lifetable |  |  |  |  |
| p_ph2d | prehypertension to death | nature mobility |  |  |  | ^10^ |
| p_h2s | hypertension to stroke | lifetable |  |  |  |  |
| p_h2m | hypertension to MI | lifetable |  |  |  |  |
| p_h2hf | hypertension to heart failure | lifetable |  |  |  |  |
| p_h2d | hypertension to death | nature mobility |  |  |  | ^10^ |
| p_s2d | stroke to death | 2.75% | 2.20% | 3.30% | Beta | ^11^ |
| p_m2d | MI to death | 9.70% | 7.76% | 11.64% | Beta | ^12^ |
| p_hf2d | Heart failure to death | 13.80% | 11.04% | 16.56% | Beta | ^13^ |
| p_ps2d | post stroke to death | 7.13% | 5.70% | 8.56% | Beta | ^11^ |
| p_pm2d | post MI to death | 6.10% | 4.88% | 7.32% | Beta | ^14^ |
| p_phf2d | post heart failure to death | 24.80% | 19.84% | 29.76% | Beta | ^15^ |
| **Cost** |  |  |  |  |  |  |
| c_uc_p | 1 year cost for prehypertension with usual care | 89.58 | 71.67 | 107.50 | gamma |  |
| c_uc_h | 1 year cost for hypertension with usual care | 91.95 | 73.56 | 110.34 | gamma |  |
| c_ls_p | 1 year cost for prehypertension with lifestyle | 268.68 | 214.95 | 322.42 | gamma |  |
| c_ls_h | 1 year cost for hypertension with lifestyle | 271.05 | 216.84 | 325.26 | gamma |  |
| c_sex_p | 1 year cost for prehypertension with strengthen exercise | 164.67 | 131.74 | 197.61 | gamma |  |
| c_sex_h | 1 year cost for hypertension with strengthen exercise | 167.04 | 133.63 | 200.44 | gamma |  |
| c_sex_s | cost that calculate only in the first year for strengthen exercise | 3923.44 | / | / | / |  |
| c_rex_p | 1 year cost for prehypertension with relaxation | 209.87 | 167.89 | 251.84 | gamma |  |
| c_rex_h | 1 year cost for hypertension with relaxation | 212.23 | 169.78 | 254.68 | gamma |  |
| c_rex_s | cost that calculate only in the first year for relaxation | 16819.53 | / | / | / |  |
| c_di_p | 1 year cost for prehypertension with diet therapy | 492.07 | 393.65 | 590.48 | gamma |  |
| c_di_h | 1 year cost for hypertension with diet therapy | 494.43 | 395.54 | 593.32 | gamma |  |
| c_di_s | cost that calculate only in the first year for diet therapy | 14119.86 | / | / | / |  |
| c_drug | cost of drugs | 84.89 | 67.91 | 101.87 | gamma | ^16^ |
| c_s | Hospitalization Cost of stroke | 4480.53 | 3584.42 | 5376.64 | gamma | ^17,18^ |
| c_m | Hospitalization Cost of MI | 4532.35 | 3625.88 | 5438.82 | gamma |  |
| c_hf | Hospitalization Cost of HF | 1300.16 | 1040.13 | 1560.19 | gamma |  |
| c_ps | Hospitalization Cost of post stroke | 448.05 | 358.44 | 537.66 | gamma |  |
| c_pm | Hospitalization Cost of post MI | 453.24 | 362.59 | 543.88 | gamma |  |
| c_phf | Hospitalization Cost of post HF | 130.02 | 104.01 | 156.02 | gamma |  |
| c_eof | Cost of end-of-life | 2179 | 1743.2 | 2614.8 | Log normal | ^19^ |
| **Utility** |  |  |  |  |  |  |
| u_ph | Utility of people with prehypertension | 0.931 | 0.931 | 1 | Beta | estimated as health people |
| u_h | Utility of people with hypertension | 0.8 | 0.64 | 0.931 | Beta | ^20^ |
| u_s | Utility of people with stroke | 0.55 | 0.44 | 0.66 | Beta |  |
| u_m | Utility of people with MI | 0.6 | 0.48 | 0.72 | Beta |  |
| u_hf | Utility of people with HF | 0.63 | 0.504 | 0.756 | Beta |  |
| u_ps | Utility of people with post stroke | 0.65 | 0.52 | 0.78 | Beta |  |
| u_pm | Utility of people with post MI | 0.7 | 0.56 | 0.84 | Beta |  |
| u_phf | Utility of people with post HF | 0.73 | 0.584 | 0.876 | Beta |  |
| **Other** |  |  |  |  |  |  |
| discount | Discount rate | 0.05 | 0.03 | 0.08 | Beta | ^21^ |
| Total_POP | Total population of China in 2021 | 14.14*10^8^ | / | / | / | ^22^ |
| POP | Population percentage of China by age group | lifetable |  |  |  | ^23^ |
| PRE_p | The prevalence of prehypertension in China | 22.14% | / | / | / | ^24^ |
| Note: MI: myocardial infarction; HF: heart failure; Unit of cost: USD. Some costs whose component including scheme design or staff training were only calculated in the first year.  Transition probabilities with no source were used through lifetable. Detailed information can be found in the supplementary material. | | | | | | |

## 4. Calculation of the Cost of Interventions

We calculated the cost of interventions by decomposing intervention measures into small items. The content and frequency of specific interventions can be found in the standardization table of interventions (Supplementary Table 8). The standardization process will refer to the original articles and National Basic Hypertension Prevention and Management Guidelines 2020 Edition^25^. For service items, it was calculated in accordance with the frequency and the cost of a single intervention. For service duration items, the human capital method combined the wages of related employees were used to calculate. For items which were neither service items nor service duration items, the calculation method was to sum up the wages of related employees and then shared by the initial input population.

### 4.1 Cost calculation of usual care

First, usual care means providing only regular blood pressure monitoring and health education without specific intervention. For usual care, people are not included in routine chronic disease management. Therefore, the cost only includes the cost of regular blood pressure monitoring and health education once every six months. Patients with BP reaching the standard are followed up at least once every three months.

Health education can be define in two types: tele-based or lecture based. The former one is usually used in routine follow-up, which conducted with high frequency. Therefore, we considered the latter one in the cost calculation of usual care. According to China's National Basic Public Health Service Subsidy Level Reference Standard (2017)^26^, the cost of lecture is $94.13 per time and an assumption of 50 people will be benefited. Thus, health education cost will be $3.77 annually. In addition, the cost of follow-up of blood pressure monitoring is $4.08 per time . Thus, regular blood pressure monitoring cost will be $16.32￥ for prehypertensive people.

### 4.2 Cost calculation of lifestyle

Lifestyle indicated that people were required to change lifestyle or lose weight according to education in addition to routine chronic disease management, regular BP monitoring and health education.

We believe that conventional chronic disease management services should be accepted by everyone, even though China has not yet included people with prehypertension^27^. According to the 2020 China Statistical Yearbook, the number of people aged 45-49 in 2019 was 95,621 (sampling rate 0.78‰)^28^. According to the 2021 China Health Statistics Yearbook, there were 610,345 management staff in 2019. According to ( 95621/0.78‰)/610345=200, it can be concluded that each management staff needs to serve about 200 patients. Since the number of input in this model is 10,000, the number of required management staff is 50.

According to He’s study^29^, the effective working hours of each management staff are 1,625 hours per year, and the annual salary is $17,678.76. Each patient need to pay $88.39 for management staff.

According to China's National Basic Public Health Service Subsidy Level Reference Standard (2017)^26^, the cost required for each person to receive hypertension management can be obtained, as shown in Supplementary Table 9.

| Supplementary Table 9 Annual cost for Early hypertension management | |
| --- | --- |
| Items | Annual cost for Early hypertension management ($) |
| Examination revealed | 1.57*4=6.28 |
| Follow up | 4.08*4=16.32 |
| Health examination | 2.67*4=10.68 |

Health education in Lifestyle was considered a tele-based one which needed a daily cost. According to China's National Basic Public Health Service Subsidy Level Reference Standard (2017)^26^, cost of daily education is $0.05/day. That is, annual cost of daily education will be $17.18.

### 4.3 Cost calculation of strengthen exercise

Strengthen exercise was considered as a specially designed strength enhancement training with the help of exercise equipment, such as isometric exercises, aerobic exercises and so on. Since exercise design and guidance all required special training for personnel, we used the one-year salary of the rehabilitation staff in the hospital to substitute the cost of training for all management staff. But we only calculated this cost once in the first year in our model^30^. Cost of management staff was calculated the same as before. We can obtain the average cost of each conduction of intervention according to Nanjing government documents. Sports equipment such as treadmills are based on the cost obtained from published studies and compared with actual conditions^31,32^. Due to the depreciation of sports equipment, the cost of sports equipment will be calculated by dividing the total cost by the total depreciation years and then dividing by 10,000 people. So that the cost each person needs to pay can be obtained. In addition, people received strengthen exercise will also be included in hypertension management.

### 4.4 Cost calculation of relaxation

Relaxation was considered to learn relaxing exercises under professional guidance, or combined with learning materials for independent exercises, which including yoga and meditation. Since such intervention also require training for management staff, the same calculation method as in strengthen exercise is adopted. The annual salary of the yoga teacher is used to substitute the cost of training^33^. The fee for each class of Yoga can be obtained according to Li's study^33^. In addition to providing yoga training, community-based chronic diseases management staff also need to protect and supervise participants. The cost estimation of Yoga mats are the same method as sports equipment^32^. Since the training materials of yoga can be produced in electronic version, the annual salary of the yoga instructor is used to calculate the cost of recording and exercise design^33^.

Patients might also receive regular traditional Chinese medicine physiotherapy for BP reduction, including acupuncture and so on. Since this highly specialized skills require professionals to perform, the wages of physiotherapists were used to replace the wages of community-based chronic disease management staff when calculating costs. The wages of physiotherapists cannot be obtained directly, so we averaged the salaries of acupuncturists to get the salary of traditional Chinese medicine physiotherapists for one year ($35612.20) according to Chen's study^34^. Hence 10,000 people each needs to pay $178.06 for 50 physiotherapists. The service prices of acupuncture (￥23/time) can be obtained through a government document from a representative city in China—Nanjing^35,36^.

For meditation, since no Chinese local cost could be found, a study from Spain was considered, of which the cost is 45.06€ (310.69￥) per participant per session^37^. Thus, a total of 8 sessions will cost participants 2485.52￥ annually. Also according to this study, cost of inviting a physiotherapist is 45.06€ (310.69￥) per appointment. Assuming a session will benefit 50 people, each patient will pay 49.71￥ for an appointment.

### 4.5 Cost of diet therapy

Diet therapy was designed by professional dietitians in accordance with the Salt reduction, DASH, alcohol reduction, and high potassium. Note that we only considered DASH here since cost calculation for other interventions were hard and DASH were representative in diet therapy. Participants follow the recipe to keep a healthy diet and receive regular supervision. Since community dietitians also need professional training, one-year salary of dietitians was used as the training cost^38^. The cost of a specific DASH was calculated based on the DASH recommended by guideline and the food prices according to the National Bureau of Statistics of China^25^. The extra cost caused by DASH were calculated by subtracting the current per capita food consumption expenditure of the Chinese from the cost of DASH^39^.

The specific cost calculation process and results are shown in Supplementary Table 10.

| Supplementary Table 10 Cost Table of Detail Items | | |
| --- | --- | --- |
| Item | Description | Cost for every Patient($) |
| ***Usual Care*** | | |
| Health education (Lecture based) | $94.13 (Benefit 50 people), 2 times a year | 3.77 |
| Regular blood pressure monitoring (Follow up) | $4.08/time | 16.32 |
| Total Cost | / | 20.08 |
| ***Lifestyle*** | | |
| Management staff | Annual salary of $17,678.76, 50 people in total | 88.39 |
| Daily education | $0.05/day, everyday | 17.18 |
| Early hypertension management | / | 37.02 |
| Total Cost |  | 142.59 |
| ***Strengthen Exercise*** | | |
| Training cost* | Annual salary of rehabilitation department | **3923.13** |
| Management staff | Annual salary of $17,678.76, 50 people in total | 88.39 |
| Isometric exercise | $3.14/time, 3 times/week, 8 weeks a year | 75.36 |
| Aerobic exercise | $4.08/time, 3 times/week, 8 weeks a year | 97.92 |
| Including whole body muscle strength training, motion training of each joint, freehand gymnastics, equipment training, gait balance function training | $2.04/time, 3 times/week, 8 weeks a year | 48.96 |
| Treadmill# | $953.41/unit, 50 units in total, 5 years depreciation | 0.95 |
| Resistance band | $4.31/unit, 200 units in total, 1 year depreciation | 0.09 |
| Early hypertension management | Prehypertension | 37.02 |
| Total Cost |  | 200.50 |
| ***Diet therapy*** | | |
| Nutrition plan and training for dietitians | Annual salary of a dietitian | **14,118.75** |
| Average annual consumption expenditure for ordinary diet | The annual per capita food consumption expenditure of Chinese residents reaches $883.38 | 883.38 |
| Average annual consumption expenditure for DASH | Average daily cost of DASH $3.77 | 1,374.23 |
| Early hypertension management | Prehypertension | 37.02 |
| Total Cost |  | 527.87 |
| ***Relaxation*** | | |
| *Yoga* |  |  |
| Yoga Training cost*# | Annual salary of a yoga teacher | **53603.83** |
| Yoga Training course production cost*# | Annual salary of a yoga teacher | **53603.83** |
| Yoga Service charge# | $15.02/class,8 class a year | 120.13 |
| Yoga mat | $14.28/unit, 200 units in total, 2 years depreciation | 0.14 |
| *Acupuncture* |  |  |
| Physiotherapist salary# | Annual salary of $35,612.20 | 178.06 |
| Acupuncture | $3.61/time, 3 times/week, 8 weeks a year | 86.64 |
| *Meditation* |  |  |
| Physiotherapist appointment | $7.80/appointment, benefit 50 people, 8 appointments in total | 9.79 |
| Meditation | $48.74/session, 8 sessions in total | 389.92 |
| Management staff | Annual salary of $17,678.76, 50 people in total | 88.39 |
| Early hypertension management | Prehypertension | 37.02 |
| Total Cost |  | 404.49 |
| ***Early hypertension management*** |  |  |
| Examination revealed | Prehypertension | 6.28 |
| Follow up | Prehypertension, every 3 months | 16.32 |
| Health examination | Prehypertension, every 3 months | 10.67 |
| Health education (Lecture based) | $14.77 (Benefit 50 people), 2 times a year | 3.77 |
| Total Cost | Prehypertension, every 3 months | 37.02 |
| ***Hypertension management*** | | |
| *The cost is only calculated once in the first year; #The cost is discounted | | |
| Examination revealed | Hypertension, every month | 18.83 |
| Follow up | Hypertension, every month | 48.95 |
| Health examination | Hypertension, every month | 32.00 |
| Health education (Lecture based) | Hypertension, every month | 103.54 |
| Total Cost |  | 203.32 |
|  | | |

## 5. Results for base-case analysis

### 5.1 Breakdown results of base-case analysis

| Supplementary Table 11 Breakdown results of the cost for base-case analysis | | | | | | | | | |
| --- | --- | --- | --- | --- | --- | --- | --- | --- | --- |
|  | c_prehypertension | c_hypertension | c_stroke | c_MI | c_HF | c_post_stroke | c_post_MI | c_post_HF | c_death |
| Non-intervention | 0.00 | 1627.00 | 392.77 | 99.32 | 55.97 | 241.56 | 69.04 | 17.40 | 579.21 |
| Usual care | 118.43 | 1610.42 | 391.36 | 98.80 | 55.86 | 240.51 | 68.63 | 17.37 | 578.81 |
| Lifestyle | 957.76 | 1469.67 | 377.99 | 94.33 | 54.96 | 230.63 | 65.12 | 17.07 | 575.27 |
| Strengthen exercise | 1446.47 | 1384.90 | 369.43 | 91.25 | 54.30 | 224.45 | 62.74 | 16.85 | 572.98 |
| Relaxation | 2893.30 | 1395.80 | 369.77 | 91.95 | 54.49 | 224.69 | 63.28 | 16.91 | 573.31 |
| Diet therapy | 3411.48 | 1513.66 | 382.51 | 95.73 | 55.24 | 233.94 | 66.21 | 17.16 | 576.40 |
|  | | | | | | | | | |
|  | | | | | | | | | |

|  | Supplementary Table 12 Breakdown results of the QALYs for base-case analysis | | | | | | | |
| --- | --- | --- | --- | --- | --- | --- | --- | --- |
|  | q_prehypertension | q_hypertension | q_stroke | q_MI | q_HF | q_post_stroke | q_post_MI | q_post_HF |
| Non-intervention | 4.47 | 6.91 | 0.05 | 0.01 | 0.03 | 0.35 | 0.11 | 0.10 |
| Usual care | 4.56 | 6.84 | 0.05 | 0.01 | 0.03 | 0.35 | 0.11 | 0.10 |
| Lifestyle | 5.32 | 6.24 | 0.05 | 0.01 | 0.03 | 0.33 | 0.10 | 0.10 |
| Strengthen exercise | 5.78 | 5.88 | 0.05 | 0.01 | 0.03 | 0.33 | 0.10 | 0.09 |
| Relaxation | 5.72 | 5.93 | 0.05 | 0.01 | 0.03 | 0.33 | 0.10 | 0.09 |
| Diet therapy | 5.08 | 6.43 | 0.05 | 0.01 | 0.03 | 0.34 | 0.10 | 0.10 |
|  |  | | | | | | | |

###
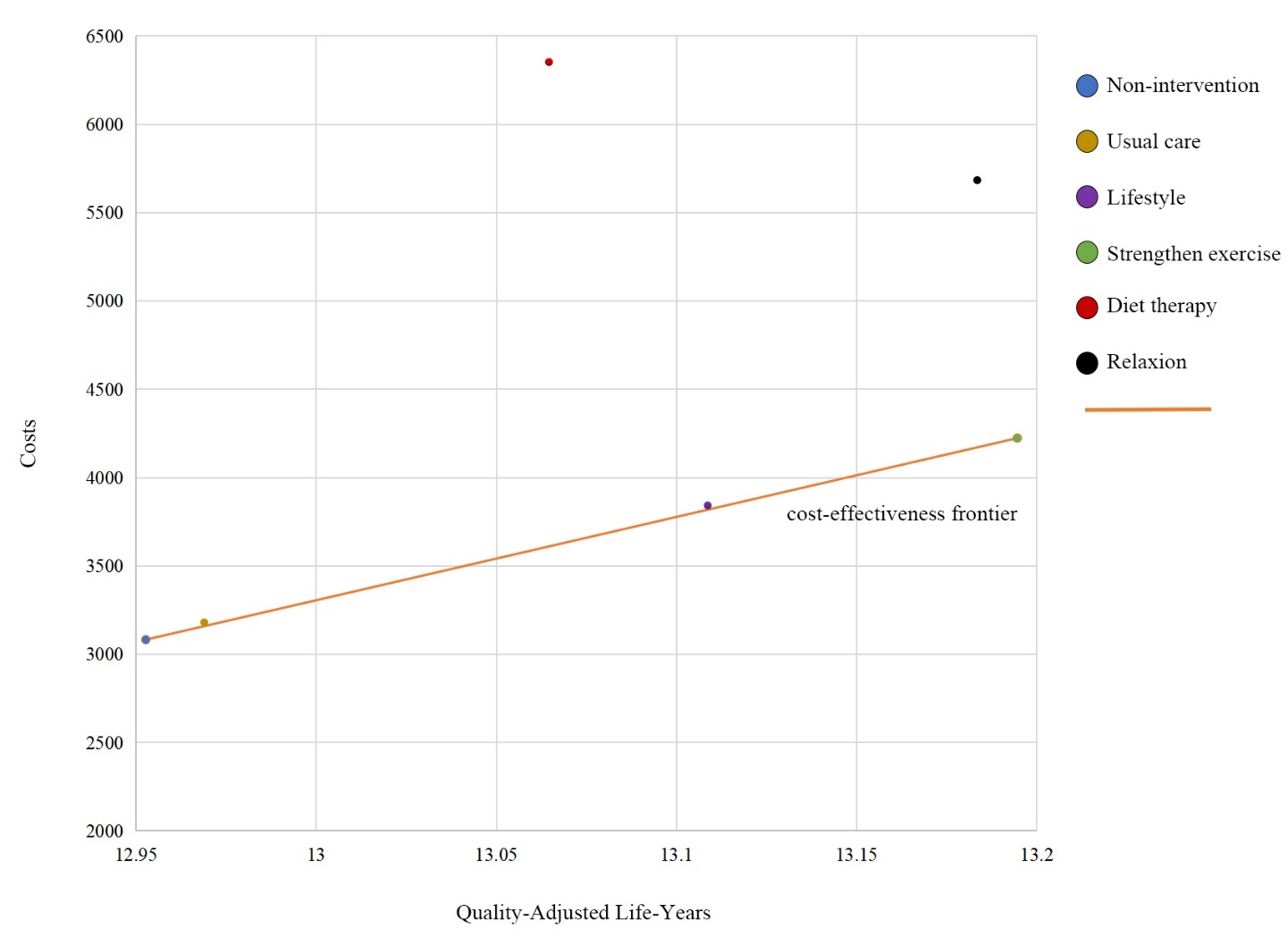
5.2 The cost-effectiveness plane of base-case analysis

Supplementary Figure 3 The cost-effectiveness plane of base-case analysis

## 6. Sensitivity Analyses Plot

### 6.1 Deterministic sensitivity analysis

| 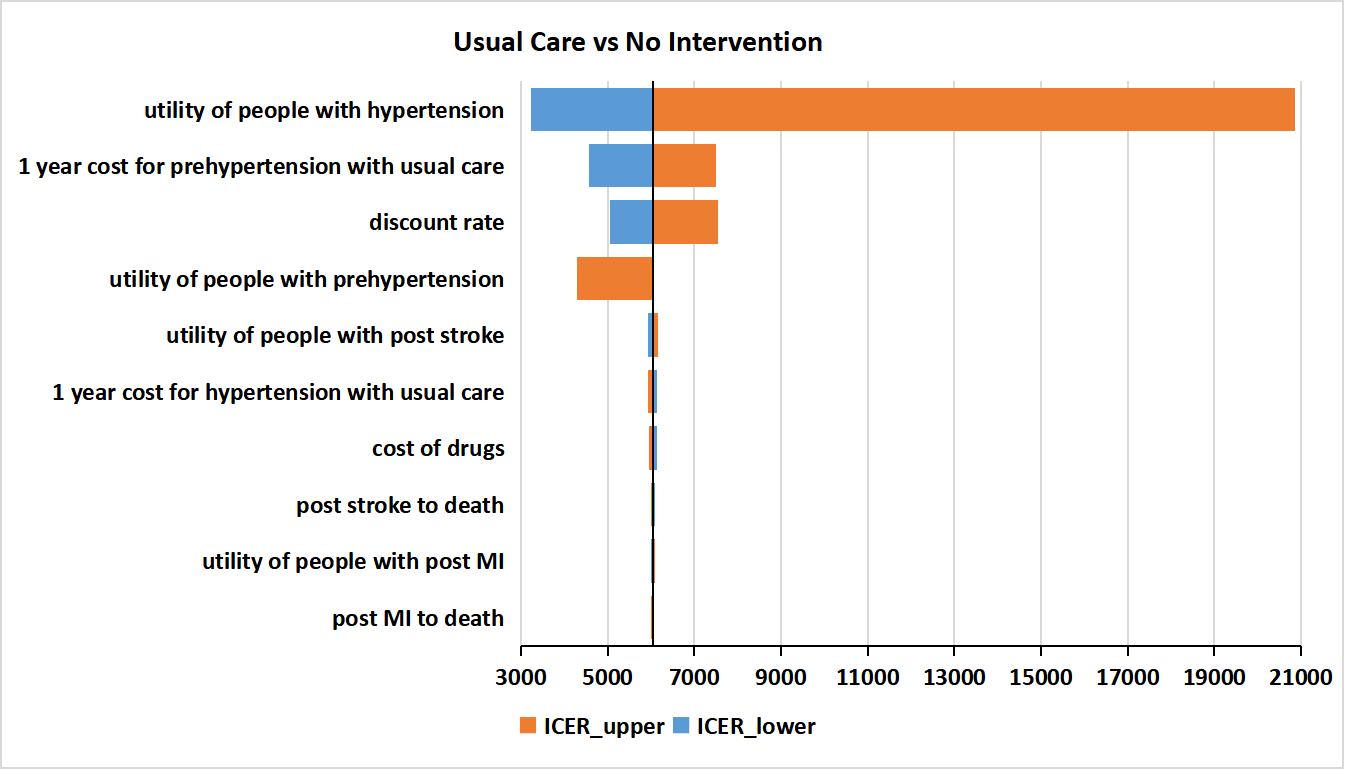 | 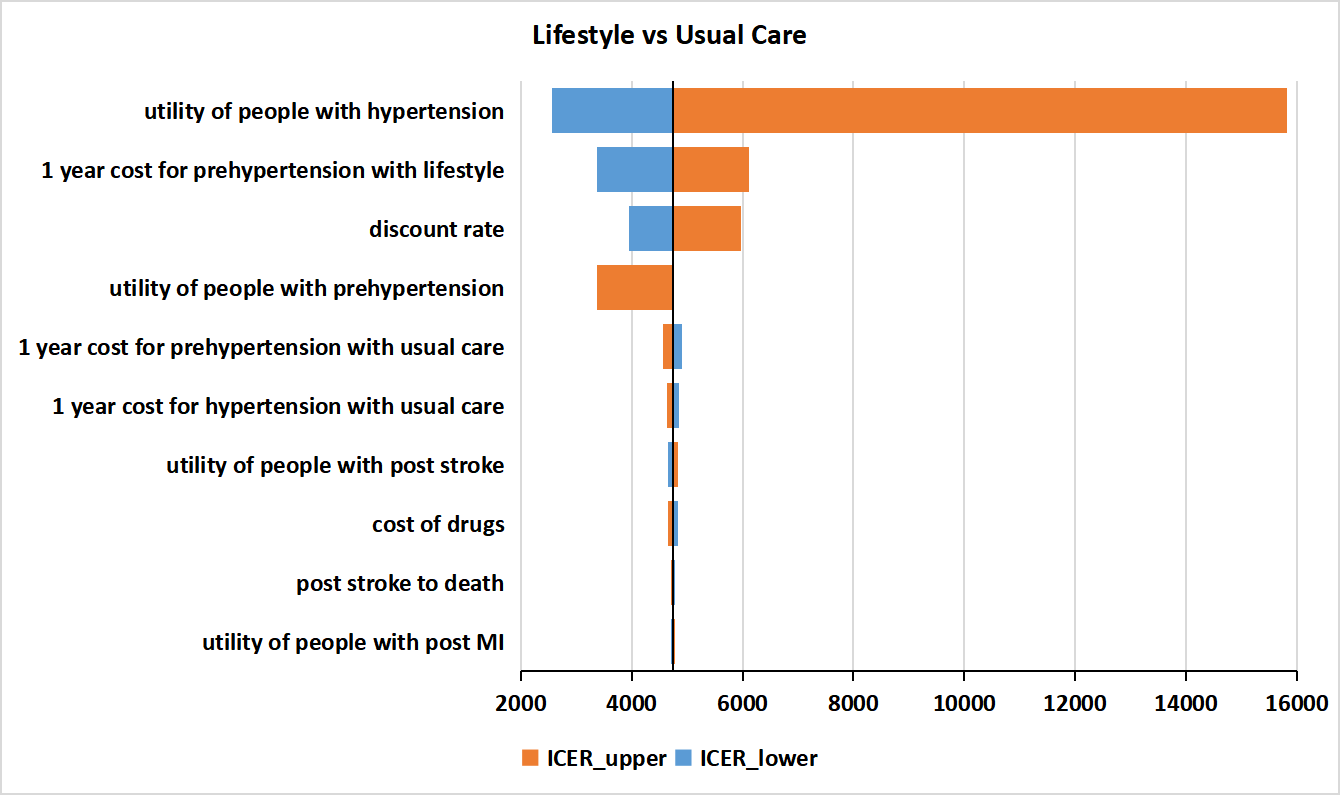 |
| --- | --- |
| 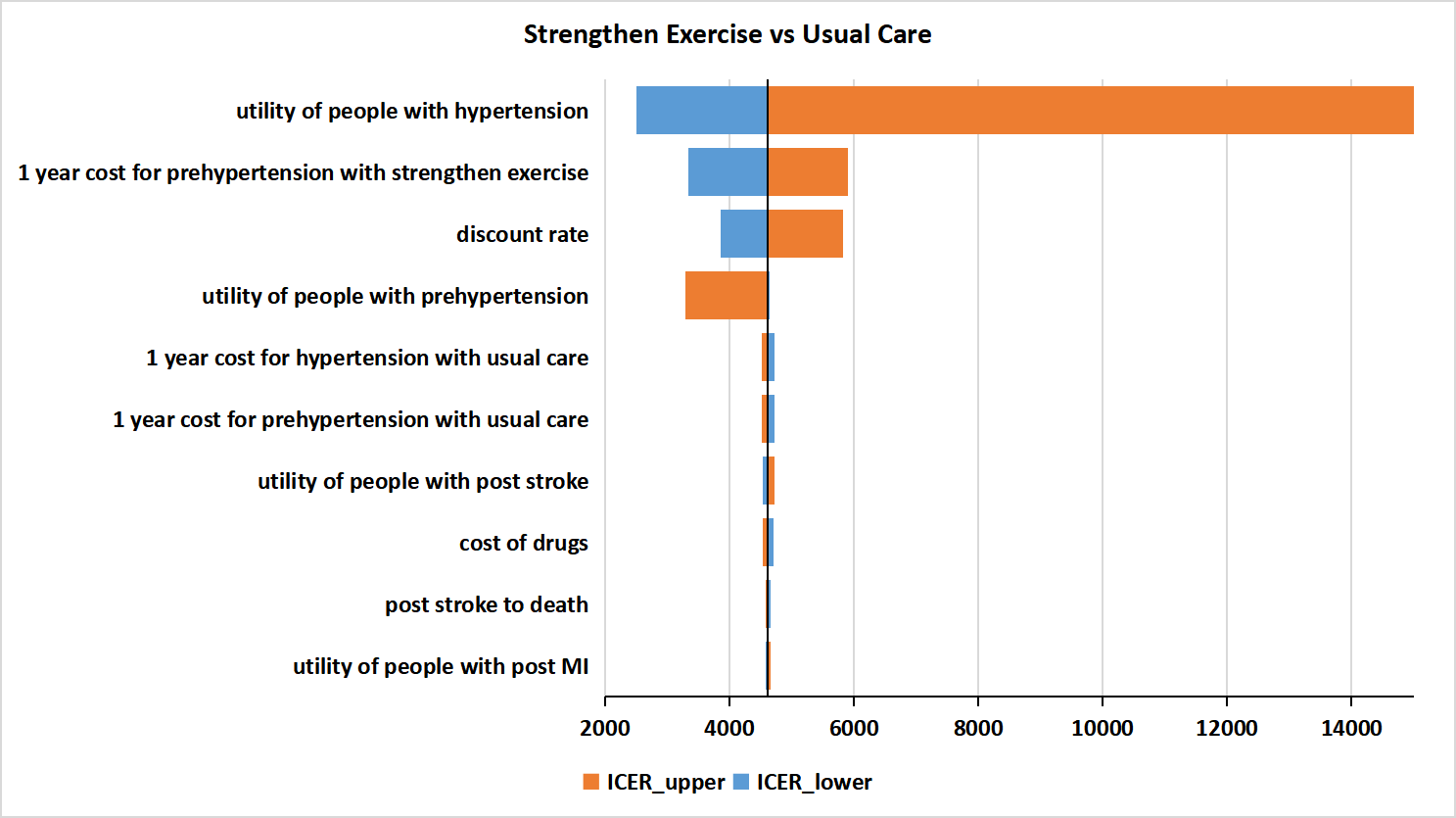 | 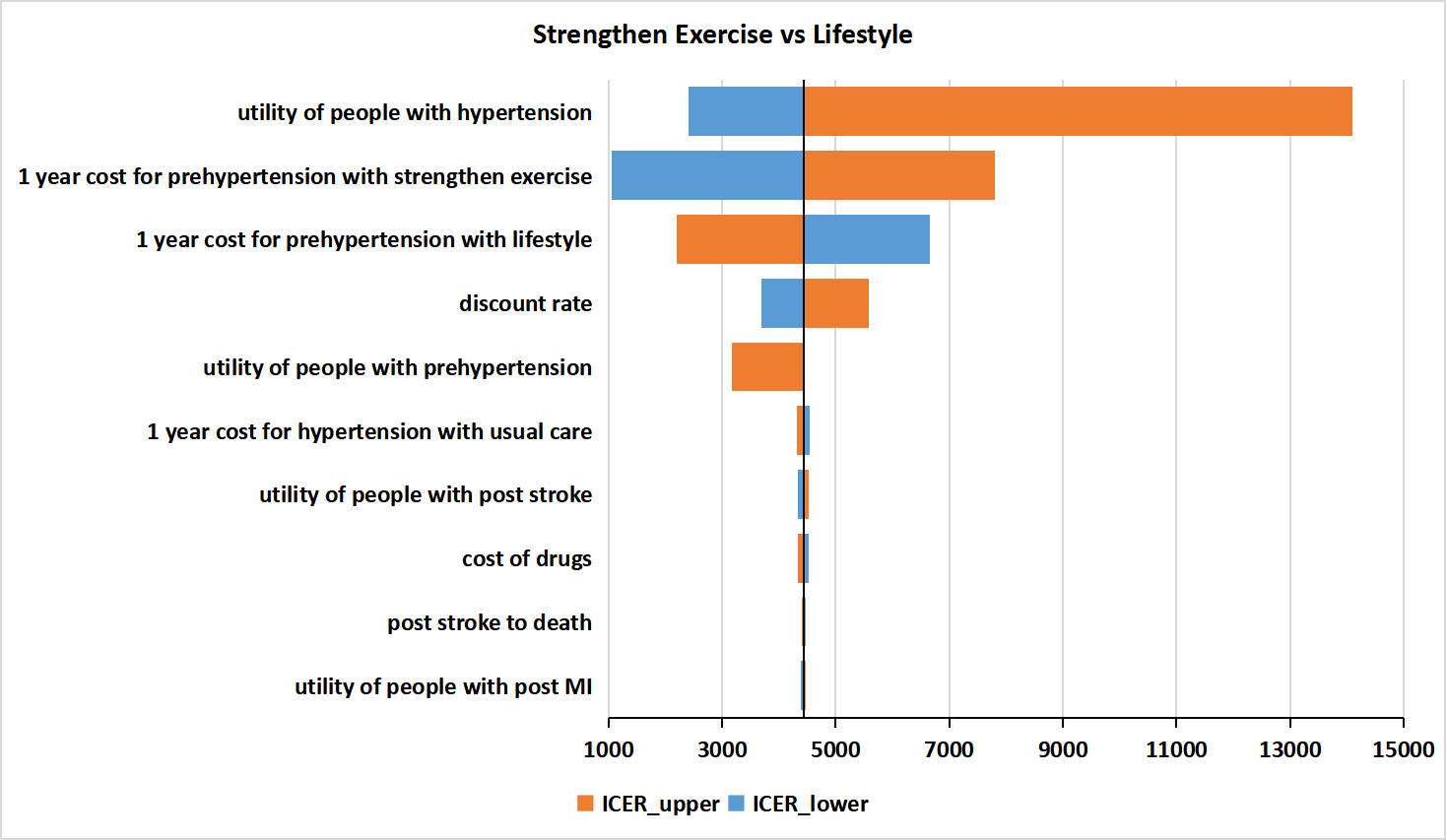 |
| Supplementary Figure 4a Tornado diagrams showing results of deterministic sensitivity | |

### 6.2 Probabilistic sensitivity analysis

| 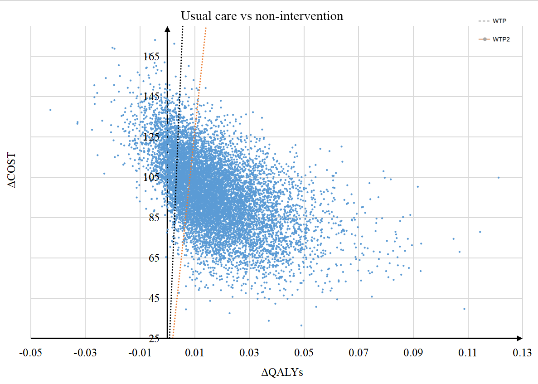 | 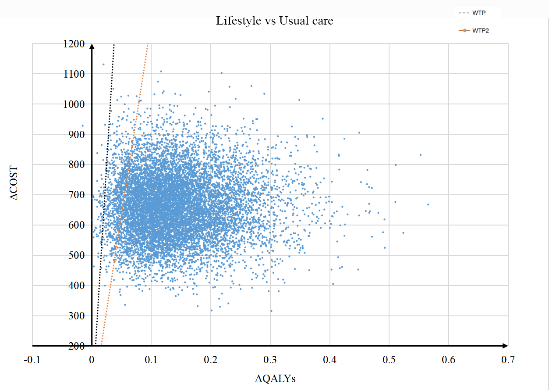 |
| --- | --- |
| 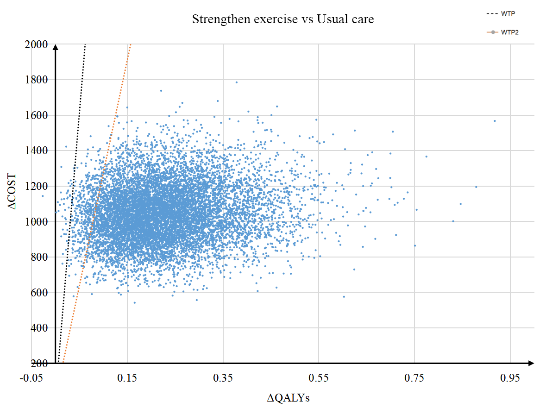 | 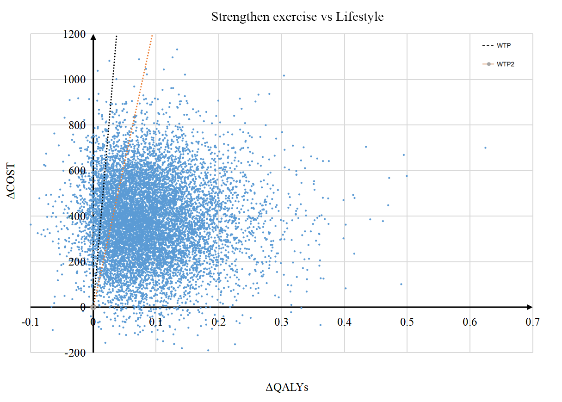 |
| Supplementary Figure 4b Probabilistic sensitivity analysis scatter plots  PSA: Probabilistic sensitivity analysis; WTP: willingness-to-pay; WTP= 12,728 $/QALY; WTP2= 38,184 $/QALY | |

## 7. Key model assumptions, methodologies and parameters input of BIA

### 7.1 Key model assumptions of BIA

(1) We assumed that the BP reduction benefits of interventions for people at all age were the same;

(2) We did not considered the costs of NPIs on hypertensive people, since management in hypertention state were not regarded as early intervention in our study;

(3) Four age groups were considered: “45-49”, “50-54”, “55-59”, “60-64”in the BIA. The lower limit of the beginning age is correspond to the CEA. The upper limit of the beginning age is based on the consideration of diagnostic criteria of hypertension in Chinese hypertension guideline. The context hypertension criteria are not applicable to people aged 65 and above, resulting in corresponding management objectives being changed.

(4) For whether to include only patients with high risk of CVE, we considered two scenarios: including all patients and including only patients with high risk of CVE.

(5) Duration of implementation varied from one year to simulation horizon.

(6) Compliance to NPIs including 100%, 80%, 60%, 40%, 20% was also considered in this model

### 7.2 Methodologies

In this BIA, we only considered one interventions with high probability to be cost-effective according to the CEAC (Figure 3 in the main text). A total four age groups were considered in this BIA. The population of specific age groups input were extracted from 2021 Chinese National Bureau of Statistics^40^. The simulation time was set as 15 years. The cost of intervention was the extra cost of the intervention for prehypertensive people, and the cost of drugs and hypertension management. The costs of interventions were used as the same value as in the cost-effectiveness analysis. The cumulative economic benefits of CVE avoided (stroke, heart failure, and myocardial infarction) were calculated. We record the annual intervention costs and economic benefits of CVE avoided for every age group. We also included scenarios of different intervention strategies, which considered four factors: (1) intervention beginning ages; (2) whether to include only patients with high risk of CVE. (3) duration of implementation. In addition, compliance to NPIs including 100%, 80%, 60%, 40%, 20% was also considered in this model

### 7.3 Parameters input

| Supplementary Table 14 Additional parameters input of the BIA | |
| --- | --- |
| Name | Value |
| Total population of China in 2021 | 1,412,600,000 |
| 45-49 age group population percent | 8.76% |
| 50-54 age group population percent | 8.53% |
| 55-59 age group population percent | 6.78% |
| 60-64 age group population percent | 5.56% |
| 65-69 age group population percent | 5.05% |
| 70-74 age group population percent | 3.27% |
| 75-79 age group population percent | 2.07% |
| 80-84 age group population percent | 1.31% |
| 85-89 age group population percent | 0.64% |
| 45-49 age group population with high risk of CVE percent | 2.70% |
| 50-54 age group population with high risk of CVE percent | 7.60% |
| 55-59 age group population with high risk of CVE percent | 7.70% |
| 60-64 age group population with high risk of CVE percent | 29.00% |
| 65-69 age group population with high risk of CVE percent | 24.40% |
| 70-74 age group population with high risk of CVE percent | 26.50% |
| 75-79 age group population with high risk of CVE percent | 50.00% |
| 80-84 age group population with high risk of CVE percent | 50.00% |
| 85-89 age group population with high risk of CVE percent | 50.00% |
| population percentage of prehypertensive people | 21.44% |
| compliof the intervention | 100.00% |

### 7.4 Scenario analyses of Budget Impact Analysis

| 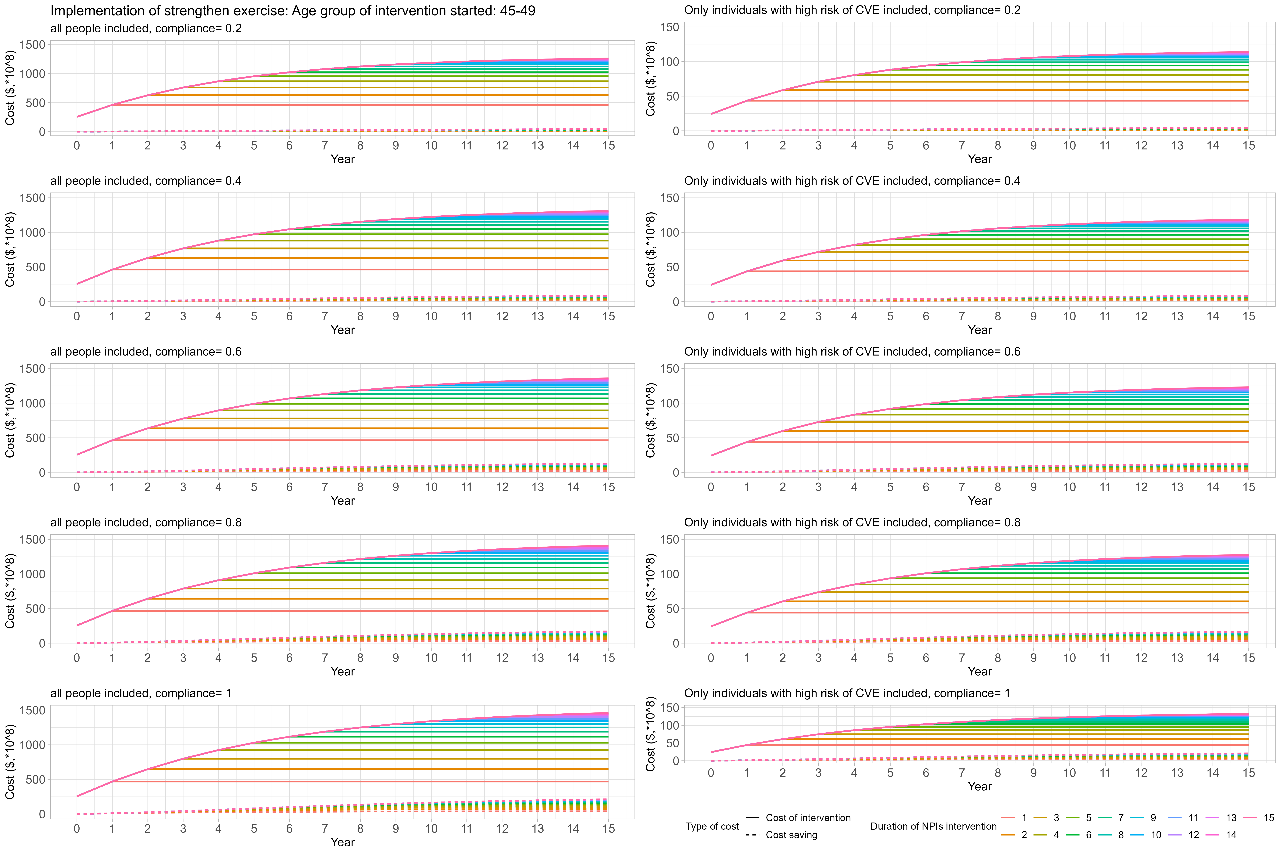 |
| --- |
| Supplementary Figure 5a Results of Scenario analyses (Age group of intervention started: 45-49) |
| 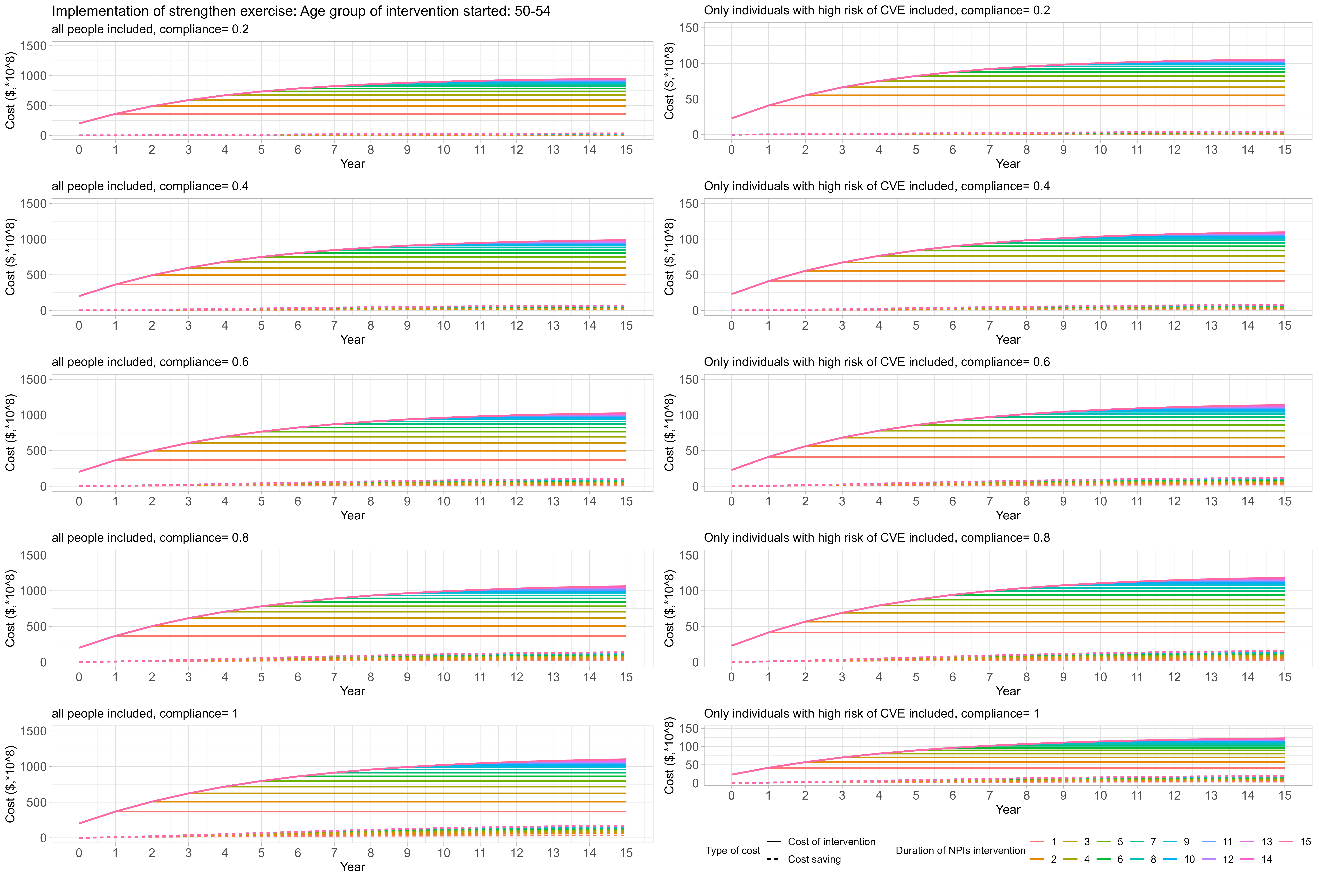 |
| Supplementary Figure 5b Results of Scenario analysis of BIA (Age group of intervention started: 50-54) |

| 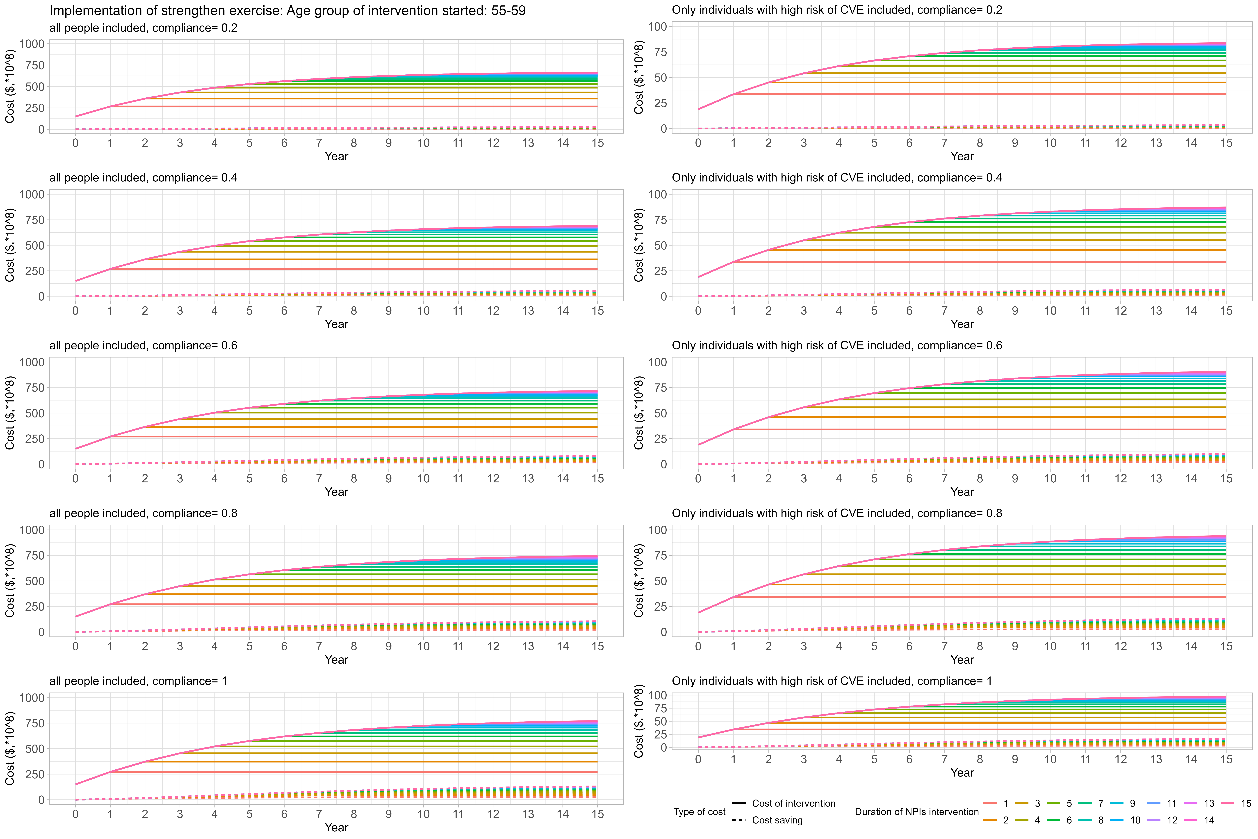 |
| --- |
| Supplementary Figure 5c Results of Scenario analyses (Age group of intervention started: 55-59) |
| 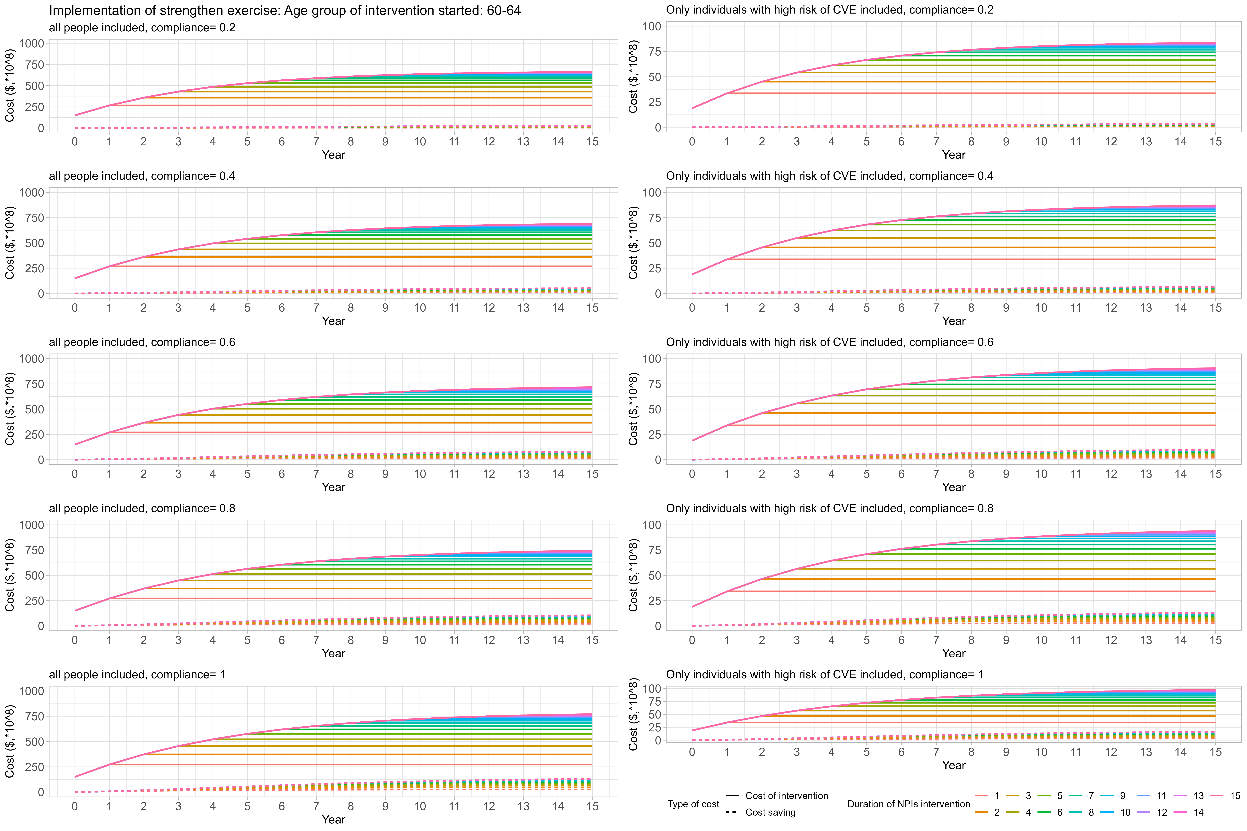 |
| Supplementary Figure 5d Results of Scenario analysis of BIA (Age group of intervention started: 60-64) |

**References**

1 Chien KL, Hsu HC, Su TC, et al. Prediction models for the risk of new-onset hypertension in ethnic chinese in taiwan*. J Hum Hypertens* 2011; **25**(5): 294-303.

2 Kannel WB, D'Agostino RB, Silbershatz H, Belanger AJ, Wilson PW, Levy D. Profile for estimating risk of heart failure*. Arch Intern Med* 1999; **159**(11): 1197-204.

3 Chien K, Su T, Hsu H, et al. Constructing the prediction model for the risk of stroke in a chinese population*. Stroke* 2010; **41**(9): 1858-64.

4 Zhang X, Attia J, D'Este C, Yu X, Wu X. A risk score predicted coronary heart disease and stroke in a chinese cohort*. J Clin Epidemiol* 2005; **58**(9): 951-8.

5 Shen Y, Chang C, Zhang J, Jiang Y, Ni B, Wang Y. Prevalence and risk factors associated with hypertension and prehypertension in a working population at high altitude in china: a cross-sectional study*. Environ Health Prev Med* 2017; **22**(1).

6 Li Y, Wu YF, Chen KP, et al. Prevalence of atrial fibrillation in china and its risk factors*. Biomed Environ Sci* 2013; **26**(9): 709-16.

7 Zhang W, Liu CY, Ji LN, Wang JG. Blood pressure and glucose control and the prevalence of albuminuria and left ventricular hypertrophy in patients with hypertension and diabetes*. The Journal of Clinical Hypertension* 2020; **22**(2): 212-20.

8 Hu L, Huang X, You C, et al. Prevalence and risk factors of prehypertension and hypertension in southern china*. Plos One* 2017; **12**(1): e170238.

9 Nkomo VT, Gardin JM, Skelton TN, Gottdiener JS, Scott CG, Enriquez-Sarano M. Burden of valvular heart diseases: a population-based study*. Lancet* 2006; **368**(9540): 1005-11.

10 National Bureau of Statistics. The sixth national census. 2010. http://www.stats.gov.cn/tjsj/pcsj/rkpc/6rp/indexch.htm (accessed 16 August, 2022).

11 Chang KC, Lee HC, Tseng MC, Huang YC. Three-year survival after first-ever ischemic stroke is predicted by initial stroke severity: a hospital-based study*. Clin Neurol Neurosurg* 2010; **112**(4): 296-301.

12 Chiang FT, Shyu KG, Wu CJ, et al. Predictors of 1-year outcomes in the taiwan acute coronary syndrome full spectrum registry*. J Formos Med Assoc* 2014; **113**(11): 794-802.

13 Lulu S, Rong L, Tao L, et al. Prognosis status with its influencing factors in heart failure patients during 1 year of discharged period*. Chinese Circulation Journal* 2013; **28**(02): 125-8.

14 Tangri N, Ferguson TW, Whitlock RH, et al. Long term health outcomes in patients with a history of myocardial infarction: a population based cohort study*. Plos One* 2017; **12**(7): e180010.

15 Kulchaitanaroaj P, Brooks JM, Chaiyakunapruk N, Goedken AM, Chrischilles EA, Carter BL. Cost-utility analysis of physician-pharmacist collaborative intervention for treating hypertension compared with usual care*. J Hypertens* 2017; **35**(1): 178-87.

16 Su M, Zhang Q, Bai X, et al. Availability, cost, and prescription patterns of antihypertensive medications in primary health care in china: a nationwide cross-sectional survey*. Lancet* 2017; **390**(10112): 2559-68.

17 Ministry of Health of the People's Republic of China. 2020 china health statistics yearbook. 2021. https://www.yearbookchina.com/navibooklist-n3020013080-1.html?from=singlemessage (accessed 21 December, 2021).

18 Wu Y, Zhou Q, Xuan J, et al. A cost-effectiveness analysis between amlodipine and angiotensin ii receptor blockers in stroke and myocardial infarction prevention among hypertension patients in china*. Value Health Reg Issues* 2013; **2**(1): 75-80.

19 Zhang L, Lin Z, Yin H, Liu J, Xuan J. Clopidogrel versus aspirin for the treatment of acute coronary syndrome after a 12-month dual antiplatelet therapy: a cost-effectiveness analysis from china payer's perspective*. Clin Ther* 2018; **40**(12): 2125-37.

20 Ren M, Xuan D, Lu Y, Fu Y, Xuan J. Economic evaluation of olmesartan/amlodipine fixed-dose combination for hypertension treatment in china*. J Med Econ* 2020; **23**(4): 394-400.

21 Guoen L. Chinese guidelines for pharmacoeconomics evaluation 2020China Market Press, 2020.

22 National Bureau of Statistics. 2020 china statistical yearbook. 2021. http://www.stats.gov.cn/tjsj/ndsj/2020/indexch.htm (accessed 21 December, 2021).

23 National Bureau of Statistics. Population by age. 2019. https://data.stats.gov.cn/easyquery.htm?cn=C01 (accessed 22 August, 2022).

24 Li W, Chen D, Liu S, et al. The rates and the determinants of hypertension according to the 2017 definition of hypertension by acc/aha and 2014 evidence-based guidelines among population aged >/=40 years old*. Glob Heart* 2021; **16**(1): 34.

25 National Cardiovascular Disease Center Office. National basic hypertension prevention and management guidelines 2020 edition*. China Recycling Magazine* 2021; **36**(3): 209-20.

26 National Health Commission of the People's Republic of China. National basic public health service projects in 2017. 2017. http://www.nhc.gov.cn/jws/s3577/201709/fb16b2e306bd469ab84e0c42173bc52d.shtml (accessed 21 December, 2021).

27 National Cardiovascular Disease Center Office. National basic hypertension prevention and management guidelines 2020 edition*. China Recycling Magazine* 2021; **36**(3): 209-20.

28 National Bureau of Statistics. 2020 china statistical yearbook. 2021. http://www.stats.gov.cn/tjsj/ndsj/2020/indexch.htm (accessed 21 December, 2021).

29 He B. Research on the current situation of national basic public health service and labor cost calculation in jiangsu province[Thesis]:Nanjing Medical University;2019.

30 Li D. Research on the application of activity-based costing in public hospital cost accounting——taking y public hospital as an example[Thesis]:Yunnan University of Finance and Economics;2021.

31 Zhang Z. Research on the design of home fitness equipment based on consumer behavior[Thesis]:Yanshan University;2014.

32 Luo L, Wang Z, Fang X, Xu H, Zhong H, Kang X. China's online sporting goods consumption status and influencing factors——an empirical analysis based on big data on tmall platform*. Journal of Shanghai Sport University* 2021; **45**(06): 35-49.

33 Li M. Empirical research on yoga movement in xi'an area——taking profitable institutions as an example[Thesis]:Xi'an Institute of Physical Education;2013.

34 Chen C. Research on perfecting the cost accounting method of traditional chinese medicine medical service in jiangsu province from the perspective of knowledge management[Thesis]:Nanjing University of Chinese Medicine;2012.

35 People's Government of Xuanwu District, Nanjing City. Announcement of the price of medical service items of primary medical and health institutions in xuanwu district (physiotherapy and rehabilitation). 2020. http://www.xwzf.gov.cn/xwqrmzf/202001/t20200107_1766768.html (accessed 21 December, 2021).

36 People's Government of Xuanwu District, Nanjing City. Announcement of the price of medical service items in primary medical and health institutions in xuanwu district (diagnosis and treatment of chinese medicine and ethnic medicine). 2020. http://www.xwzf.gov.cn/xwqrmzf/202001/t20200107_1766775.html (accessed 21 December, 2021).

37 Perez-Aranda A, D'Amico F, Feliu-Soler A, et al. Cost-utility of mindfulness-based stress reduction for fibromyalgia versus a multicomponent intervention and usual care: a 12-month randomized controlled trial (eudaimon study)*. J Clin Med* 2019; **8**(7).

38 Xian X. Research on marketing strategy of jh vocational training company[Thesis]:Guangxi Normal University;2016.

39 National Bureau of Statistics. 2020 china statistical yearbook. 2021. http://www.stats.gov.cn/tjsj/ndsj/2020/indexch.htm (accessed 21 December, 2021).

40 National Bureau of Statistics. Population by age. 2019. https://data.stats.gov.cn/easyquery.htm?cn=C01 (accessed 22 August, 2022).
